# Motor Learning Requires Purkinje Cell Synaptic Potentiation through Activation of AMPA-Receptor Subunit GluA3

**DOI:** 10.1016/j.neuron.2016.11.046

**Published:** 2017-01-18

**Authors:** Nicolas Gutierrez-Castellanos, Carla M. Da Silva-Matos, Kuikui Zhou, Cathrin B. Canto, Maria C. Renner, Linda M.C. Koene, Ozgecan Ozyildirim, Rolf Sprengel, Helmut W. Kessels, Chris I. De Zeeuw

**Affiliations:** 1 Synaptic Plasticity and Behavior Group, The Netherlands Institute for Neuroscience, Royal Netherlands Academy of Arts and Sciences, 1105 BA Amsterdam, the Netherlands; 2 Cerebellar Coordination and Cognition Group, The Netherlands Institute for Neuroscience, Royal Netherlands Academy of Arts and Sciences, 1105 BA Amsterdam, the Netherlands; 3 Department of Neuroscience, Erasmus MC Rotterdam, 3015 GE Rotterdam, the Netherlands; 4 Max Planck Institute for Medical Research, 69120 Heidelberg, Germany

**Keywords:** learning, cerebellum, Purkinje cell, synapse, LTP, AMPA receptor, GluA3, Epac

## Abstract

Accumulating evidence indicates that cerebellar long-term potentiation (LTP) is necessary for procedural learning. However, little is known about its underlying molecular mechanisms. Whereas AMPA receptor (AMPAR) subunit rules for synaptic plasticity have been extensively studied in relation to declarative learning, it is unclear whether these rules apply to cerebellum-dependent motor learning. Here we show that LTP at the parallel-fiber-to-Purkinje-cell synapse and adaptation of the vestibulo-ocular reflex depend not on GluA1- but on GluA3-containing AMPARs. In contrast to the classic form of LTP implicated in declarative memory formation, this form of LTP does not require GluA1-AMPAR trafficking but rather requires changes in open-channel probability of GluA3-AMPARs mediated by cAMP signaling and activation of the protein directly activated by cAMP (Epac). We conclude that vestibulo-cerebellar motor learning is the first form of memory acquisition shown to depend on GluA3-dependent synaptic potentiation by increasing single-channel conductance.

## Highlights

• Cerebellar learning depends on expression of GluA3, but not GluA1, in Purkinje cells
• GluA3 is required to induce LTP, but not LTD, at PF-PC synapses
• GluA3-dependent potentiation involves a cAMP-driven change in channel conductance
• GluA3-mediated LTP and learning are induced via cAMP-mediated Epac activation

## Teaser

Gutierrez et al. show a novel form of synaptic plasticity that entails an increase in channel conductance of GluA3-containing AMPARs and that is required for vestibulo-cerebellar motor learning.

## Introduction

Plasticity mediated by synaptic trafficking of α-amino-3-hydroxy-5-methyl-4-isoxazolepropionic-acid-type glutamate receptors (AMPARs) plays an important role in the acquisition of declarative memories (Kessels and Malinow, 2009). Ionotropic AMPARs drive fast, excitatory neuronal activity and can consist of four different subunits named GluA1 through GluA4. In hippocampal pyramidal cells, most AMPARs are hetero-oligomers composed of either GluA1/GluA2 or GluA2/GluA3 subunits, and the subunit composition dictates which role AMPARs play in synaptic plasticity (Shi et al., 2001). In the hippocampus, cortex, and amygdala, both long-term potentiation (LTP) and learning depend on the trafficking of GluA1-containing AMPARs to synapses (Makino and Malinow, 2011, Nedelescu et al., 2010, Rumpel et al., 2005, Mitsushima et al., 2011), whereas GluA3-containing AMPARs contribute relatively little to synaptic currents, synaptic plasticity, or learning (Adamczyk et al., 2012, Meng et al., 2003, Humeau et al., 2007). To what extent GluA1 and GluA3 play a role in adaptive motor behavior remains to be established.

Here we sought to unravel the potential role of GluA1- and/or GluA3-containing AMPARs in cerebellar motor learning. Unlike the rich insight into the role of AMPARs in declarative memory formation in the hippocampus, relatively little is known about their role in procedural memory formation in the cerebellum. AMPAR plasticity occurs at parallel-fiber-to-Purkinje-cell (PF-PC) synapses, reflecting the expression of LTP or long-term depression (LTD) (Kakegawa and Yuzaki, 2005, Steinberg et al., 2006), but the full functional significance of this plasticity and the precise molecular pathways underlying it remain to be further elucidated (Gao et al., 2012). In addition, the roles of GluA1- and/or GluA3-containing AMPARs in the plasticity of Purkinje cells (PCs) have hardly been studied (Bats et al., 2013, Douyard et al., 2007, Kakegawa and Yuzaki, 2005).

We found that adaptation of compensatory eye movements, which is one of the most widely studied forms of cerebellar motor learning serving to stabilize gaze (Anzai et al., 2010, Nguyen-Vu et al., 2013, Schonewille et al., 2011), depends on GluA3-containing AMPARs, but not on GluA1-containing AMPARs. The GluA3-containing AMPARs in PCs are critical for the induction and expression of PF-PC LTP not by trafficking of receptors, but by a change in the conductance and open probability of the channel. This form of plasticity requires activation of Epac through an increase of cyclic AMP. Together, these findings not only show that GluA3 is crucial for cerebellar potentiation and learning, but also that its actions of plasticity are evoked through a novel mechanism.

## Results

### Cerebellar Motor Learning Depends on GluA3, but Not on GluA1

**Figure 1:**
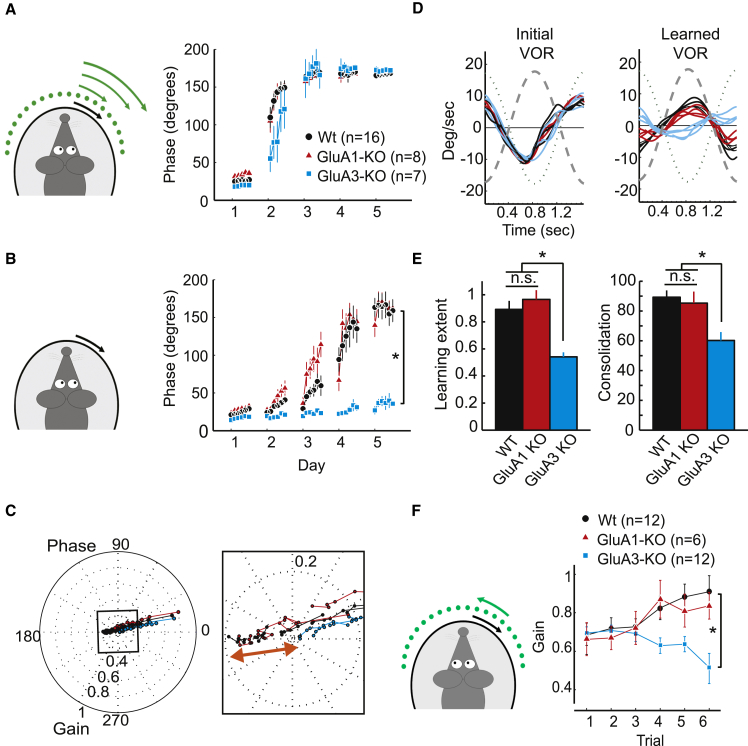
GluA3 Is Required for Oculomotor Learning (A) When adult (3–5 months of age) wild-type (WT) (black), GluA1-KO (red), and GluA3-KO (blue) mice are subjected to a visuovestibular mismatch training paradigm in which the visual screen rotates sinusoidally in the same direction as the turntable but at an increasingly greater amplitude (also referred to as a phase-reversal task), they show a similar ability to follow the training signal over time as long as the light is on. Eye movement signals are expressed as phase values (in degrees) with respect to those of the turntable, which also rotates in a sinusoidal fashion (i.e., 360° represents one sinusoidal cycle). (B) However, when the light is turned off but the turntable stimulus continues (i.e., the VOR-adaptation catch trials of the phase-reversal task), the phase values of the GluA3-KO mice show significantly impaired motor learning compared to those of GluA1-KO and WT mice. (C) Polar plot showing the trajectory of VOR gain and phase change during adaptation for WT (black line), GluA3-KO (blue), and GluA1-KO (red) mice. Gain (i.e., amplitude of the eye movement divided by that of the stimulus) is represented as distance from the center, and phase is represented as the angle relative to perfect compensation at 0°. The data reveal a common learning trajectory and comparable initial gain but a difference in learning extent between the groups. Inset shows the final VOR reached after 5 days of training, amplified to visualize the magnitude of the gain difference (red arrow) between the groups tested. (D) GluA3-KO mice (blue line) were unable to reverse their VOR phase, unlike WT (black) and GluA1-KO (red) mice. Four representative eye-velocity traces per group compare the initial VOR before (left) and after (right) the mismatch training (left). (E) Both learning extent and consolidation during the phase-reversal task are significantly smaller in GluA3-KO mice than in WT and GluA1-KO mice (T2 test p < 0.05). (F) Gain-increase learning also reveals deficits in GluA3-KO mice, but not in GluA1-KO mice, as compared to WT mice. Error bars indicate SEM; ^∗^ indicates p < 0.05.

Unlike GluA2 global knockout (KO) mice, which suffer from severe motor performance deficits including ataxia (Gerlai et al., 1998, Jia et al., 1996), mice that lack AMPAR subunit GluA1 or GluA3 (GluA1-KO and GluA3-KO) displayed intact basic motor behavior (Figure S1). Indeed, they were able to stabilize the images on their retina and/or gaze with respect to a moving visual field (i.e., optokinetic reflex, or OKR; Figure S1A), with respect to their head movements (i.e., the vestibulo-ocular reflex in the dark, or VOR: Figure S1B), or with respect to a combination of both, as occurs in daily life (i.e., VOR in the light, or VORL; Figure S1C). None of the comparisons between GluA1-KO mice, GluA3-KO mice, and their control littermates showed a significant difference in any of these paradigms (for p values, see Figure S1 and corresponding legends). Far more challenging is the test for VOR phase-reversal adaptation, which involves cerebellum-dependent motor learning (Gutierrez-Castellanos et al., 2013). During this paradigm, mice learn to shift the phase of their VOR following sinusoidal visuovestibular mismatch stimulation, in which the visual stimulus moves in the same direction as the vestibular stimulus (i.e., in phase), yet at a greater amplitude (Figure 1A). After 5 days of visuovestibular training, wild-type (WT) mice moved their eyes during table stimulation in the dark in the same direction as the body, rather than the opposite direction as they used to do before the training (i.e., they normally show an innate contraversive compensation). More specifically, the mature WT mice learned to shift their VOR in the dark by 159° out of the perfect 180° after the training (Figure 1B). Likewise, when we subjected littermate GluA1-deficient mice to this phase-reversal adaptation paradigm, they reached final average phase shifts of 162° (GluA1-KO versus WT, p = 0.13; Figure 1B), indicating that GluA1-containing AMPARs are dispensable for VOR adaptation. In contrast, GluA3-deficient mice showed striking deficits in shifting the phase of their VOR in the dark; they showed a final phase shift of only 35° after five training sessions (GluA3-KO versus WT, p = 0.001; Figure 1B). When we looked not only at the oculomotor phase but also at the learning trajectory extent (as explained in Figure S2A), we observed that, although the initial performances of VOR catch-learning trials were not significantly different for any of the three groups tested (p = 0.3 and p = 0.11 for GluA1-KO and GluA3-KO, respectively), the final performances of GluA3-KO after 5 days of training were significantly different from those of both WT littermates (p = 0.01) and GluA1-KO mice (p = 0.01) (Figure 1C). Accordingly, the vector of total learning extent per mouse, which equals the distance between the initial (first recording, day 1) and the final VOR performance coordinate throughout the 5-day-spanning phase-reversal paradigm (Figure S2), was significantly smaller for GluA3-KO mice than it was for WT and GluA1-KO mice (p = 0.001 and p = 0.0002, respectively) (Figures 1D and 1E). Moreover, the consolidation rate of learning, which equals the ratio between the total learning extent and the ideal learning extent with no overnight memory loss between training days (Figure S2A), was also significantly lower in GluA3-KO than in both GluA1-KO and WT littermates (p = 0.0008 and p = 0.001, respectively) (Figure 1E). Importantly, all groups of mice performed equally well during the visually driven vestibular training trials over the 5 days of training (all p values > 0.05; Figure 1A), indicating that the learning deficits in GluA3-KO as measured in the dark during the catch trials did not directly result from a poor response to the visuovestibular training signal but rather from an impaired ability to maintain this learned vestibular response in the absence of the visual cue (Figure 1B).

In addition to phase modulation, we also investigated gain modulation of vestibulo-ocular movements after either in-phase (gain-down) or out-of-phase (gain-up) visuovestibular training paradigms that aim to reduce or increase the amplitude, respectively, of the eye-movement response to a constant vestibular input. GluA3-KO mice showed severe learning deficits in both the gain-down (p = 0.001 for final catch trials) and gain-up (p = 0.009 for final catch trials) paradigms, whereas GluA1-KO and WT mice again performed equally well in both training paradigms (p = 0.11 and p = 0.2 for gain-down and gain-up final catch trials, respectively) (Figures 1F and S1D). These experiments indicate that GluA3-containing AMPARs contribute to cerebellum-dependent motor learning.

### GluA3 Is Required to Induce LTP, but Not LTD, at PF-PC Synapses

**Figure 2:**
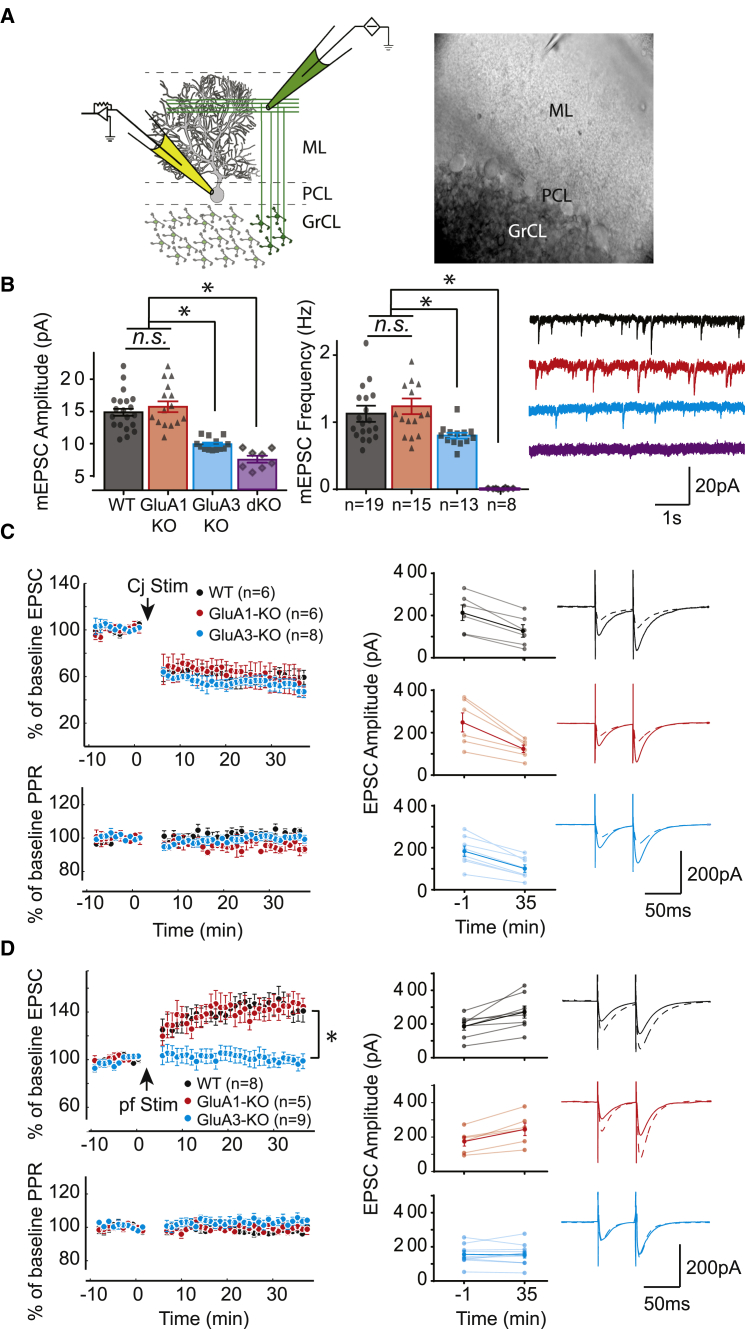
GluA3 Is Required for PF-PC LTP, but Not LTD (A) Scheme of cerebellar cortical circuitry (left) and representative picture of the in vitro preparation (right) showing positions of recording electrode (yellow) at PC soma and stimulus electrode (green) at parallel fiber beam. ML, PCL, and GrCL indicate molecular layer, PC layer, and granule cell layer, respectively. (B) mEPSC amplitude (left) and frequency (middle) of both single GluA3-KO PCs (blue bar) and double GluA1/GluA3-KO PCs (purple bar) were significantly reduced compared to those in WT PCs (black bar) (for amplitude and frequency, WT versus GluA3-KO, p = 0.0003 and p = 0.023, respectively; for WT versus GluA1&3-dKO, p < 0.0001 and p < 0.0001, respectively) and single GluA1-KO PCs (red bar) (for amplitude and frequency, GluA1-KO versus GluA3-KO, p < 0.0001 and p = 0.0032, respectively). In contrast, GluA1-KO and WT PCs presented comparable basal transmission (for amplitude and frequency, WT versus GluA1-KO, p = 0.37 and p = 0.16, respectively). Right panel shows corresponding raw traces of mEPSCs. (C) Both GluA1-KO (red) and GluA3-KO (blue) mice show similar cerebellar synaptic weakening after LTD induction compared to WT littermates (black) (top left) with unchanged PPR over time (bottom left). EPSC magnitude was held in a comparable range for all cases to prevent potential bias due to differential basal synaptic strength (middle). Representative traces are of paired EPSCs before (solid lines) and after (dashed lines) LTD induction (right, matched genotype color code). Cj Stim indicates conjunctive stimulation (so as to induce LTD). (D) GluA3-KO PCs show severe deficits in PF-PC LTP compared with WT and GluA1-KO PCs with no changes in PPR or baseline EPSC magnitude. Representative traces of paired EPSCs before (solid lines) and after (dashed lines) LTP induction (same configuration as in B). pf Stim indicates parallel-fiber-only stimulation (so as to induce LTP). Error bars indicate SEM; ^∗^ indicates p < 0.05.

PCs form the sole output of the cerebellar cortex. It has previously been shown that synaptic plasticity at their parallel fiber afferents crucially contributes to motor learning (Schonewille et al., 2010). To investigate the contribution of GluA1- and GluA3-containing AMPARs to basal synaptic transmission, we recorded spontaneous miniature excitatory synaptic currents (mEPSCs) of PCs in cerebellar slices from 4–6 week old mice (Figure 2A), an age at which GluA3-KO mice showed motor learning deficits similar to those shown during adulthood (Figure S2D). The average amplitude and frequency of mEPSCs in GluA1-deficient PCs were not significantly different (p = 0.4 and p = 0.2, respectively) from those in WT PCs (Figure 2B). In PCs of GluA3-KO mice, the average amplitude (p = 0.0003) and frequency (p = 0.02) of mEPSCs were significantly lower than those in WT PCs. The low basal transmission in the GluA3-KO mice was neither reflected in structural changes at the level of spine densities of proximal or distal PC dendrites (p = 0.7 and p = 0.2 for proximal and distal, respectively) (Figure S3) nor compensated for by increased synaptic currents from kainate receptors (for details, see Figure S4 and corresponding legends). In PCs of mice that lack both GluA1 and GluA3, mEPSC events were virtually absent (Figure 2B), suggesting that the large majority of synaptic currents in PCs are derived from either GluA1- or GluA3-containing AMPARs.

A reduced basal transmission in GluA3-deficient PC synapses can either be a cause or a consequence of impaired synaptic plasticity. We therefore investigated both LTD and LTP at the PF-PC synapse using whole-cell recordings. LTD was induced either by pairing PF stimulation with a depolarizing voltage-clamp step, mimicking climbing fiber (CF) input (Linden, 2001; Figure 2C), or by pairing PF stimulation with CF stimulation (Schonewille et al., 2011; Figures S3C–S3E). The magnitudes of LTD in PCs of GluA1-KO and GluA3-KO mice were indistinguishable from those in the PCs of WT littermates with either induction protocol (with somatic depolarization for GluA1-KO versus WT, p = 0.4, and for GluA3-KO versus WT, p = 0.2; with direct CF stimulation for GluA1-KO versus WT, p = 0.9, and for GluA3-KO versus WT, p = 0.8). These data are in line with other studies showing that GluA2 is the key subunit for AMPAR internalization and therefore for LTD induction (Steinberg et al., 2006, Schonewille et al., 2011). Next, we induced LTP in PCs by 1 Hz tetanic stimulation of PF input alone (Lev-Ram et al., 2002) (Figure 2D). This stimulus protocol reliably produced significant LTP in both WT PCs (p = 0.0005) and GluA1-KO PCs (p = 0.0003) with a similar magnitude (p = 0.5) and without significant changes in paired-pulse facilitation (PPF) of the evoked EPSCs after LTP induction (p = 0.11 and p = 0.6 in GluA1-KO and WT PCs, respectively). In contrast, with the same stimulation protocol, LTP could not be induced in GluA3-KO PCs (p = 0.7) (Figure 2D). These experiments demonstrate that PF-PC LTP requires GluA3-containing AMPARs.

### GluA3-Dependent Synaptic Potentiation Involves a cAMP-Driven Change in Channel Conductance

**Figure 3:**
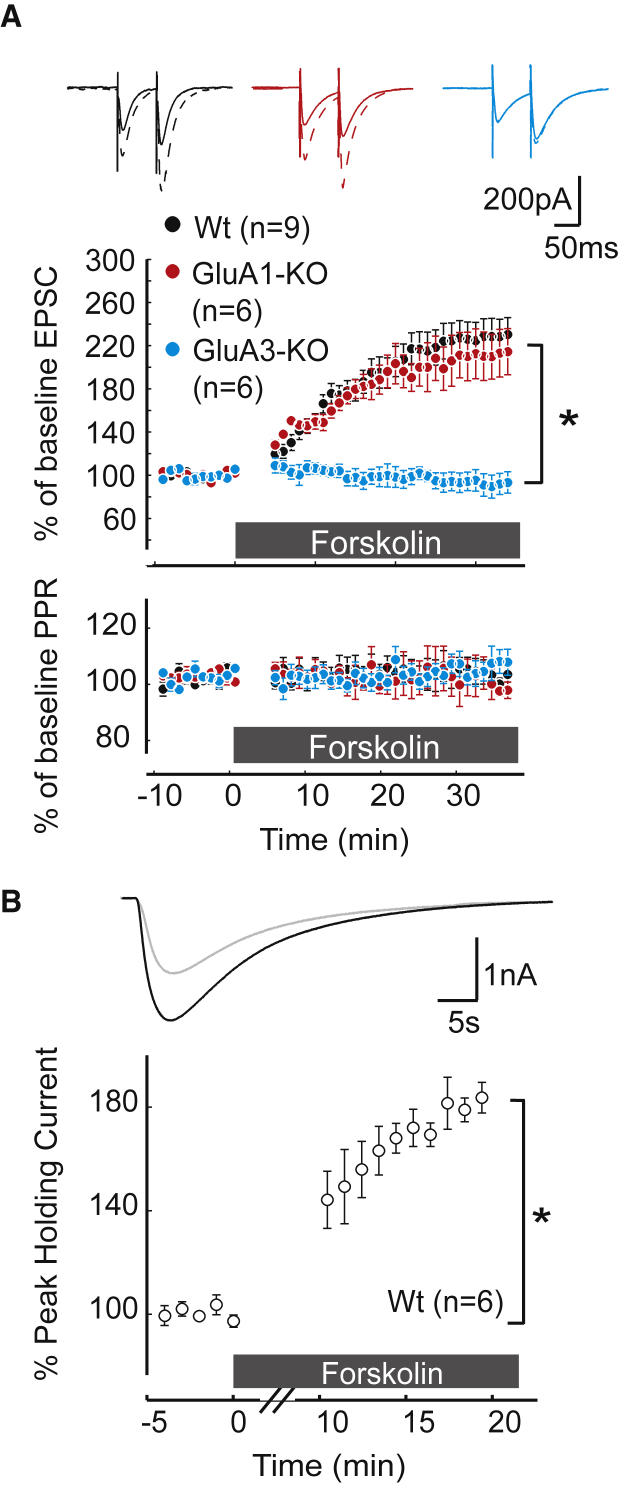
Rising cAMP Levels Produce GluA3-Dependent Postsynaptic Potentiation (A) Wash-in of 50 μM FSK causes synaptic potentiation at WT PCs (black) and GluA1-KO PCs (red), but not at GluA3-KO PCs (blue). Top, middle, and bottom show example traces, normalized EPSC amplitude, and paired pulsed ratio (PPR), respectively. (B) Enhancement of currents evoked by local puffs of 1 μM AMPA at the molecular layer following FSK application can also occur in the presence of TTX-blocking PF input. Error bars indicate SEM; ^∗^ indicates p < 0.05.

What is the molecular mechanism underlying GluA3-dependent LTP of PF-PC synapses? To test whether GluA3-dependent synaptic plasticity in PCs depends on cAMP signaling, we administered the adenylyl cyclase activator forskolin (FSK) to PCs of GluA3-KO brain slices and compared the effects to those in WT slices and GluA1-KO slices (Figure 3A). Whereas FSK produced, on average, a 2-fold potentiation in PF-evoked EPSCs in both WT and GluA1-KO PCs (230% ± 25% and 215% ± 35%, respectively), it failed to induce synaptic potentiation in PCs that lack GluA3 (95% ± 10%, p = 0.005, GluA3-KO versus WT). Importantly, AMPAR potentiation also occurred in WT PCs when local stimulation with 1 μM AMPA was used while blocking PFs with TTX (189% ± 17%, p = 0.001; Figure 3B), highlighting its postsynaptic nature (Chen and Regehr, 1997). These data indicate that GluA3-dependent synaptic potentiation at PF-PC synapses can occur upon a rise in the cellular level of cAMP.

**Figure 4:**
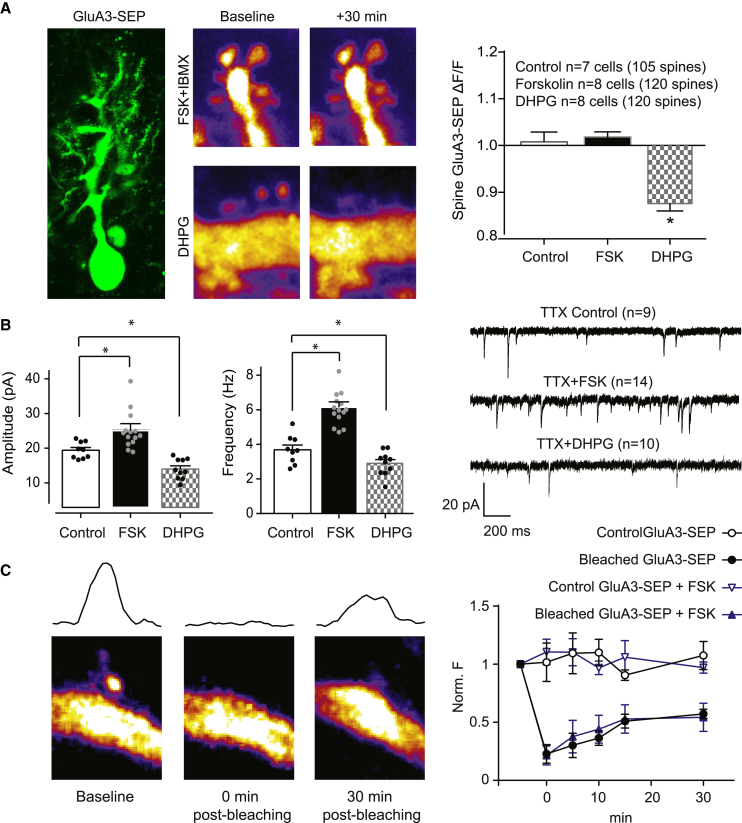
Rising cAMP Levels Produce GluA3-Dependent Synaptic Potentiation without AMPAR Trafficking (A) Left column is a *Z*_max_ projection of a stack of pictures showing a representative GluA3-SEP-transfected PC. In the top row, example pictures of a PC dendrite expressing GluA3-SEP before (middle) and after (right) FSK application were color-coded according to the fluorescence intensity to improve the visualization of, in this case, the absence of changes of surface GluA3-SEP over time. In the bottom row, example pictures of a PC dendrite expressing GluA3-SEP before (middle) and after (right) DHPG application reveal a significant reduction in synaptic GluA3-SEP over time. The right column shows that fluorescence intensity after FSK application, normalized by the fluorescence before application (FSK, middle bar), showed no significant increase of GuA3-SEP compared to the spines in which the drug was not applied (control, left bar). However, DHPG application significantly reduced GluA3-SEP in PC spines in accordance with the observed synaptic depression. (B) Despite the lack of a detectable increase in surface GluA3-SEP, FSK produced a significant increase in mEPSC amplitude and frequency in GluA3-SEP-transfected PCs in organotypic slices. DHPG induced a significant decrease in mEPSC amplitude and frequency in these neurons. (C) On the left is an example baseline maximum intensity projection z stack (3 μM, six optical planes) of a dendrite transfected with GluA3-SEP obtained with two-photon microscopy before, immediately after, and 30 min after photobleaching of the spine. The black traces above the pictures represent quantifications of SEP fluorescence across the spine and parallel to the dendrite. On the right is an overall quantification of spine FRAP dynamics over time for PCs transfected with GluA3-SEP, either with (n = 5) or without (n = 4) 50 μM FSK added after the moment of bleaching (0 min). SEP fluorescence intensity is normalized to baseline intensity (−5 min). No changes in SEP intensity were observed over time in spines neighboring the bleached spines.

We next examined whether cAMP-driven synaptic potentiation is a result of synaptic trafficking of GluA3-containing AMPARs. To assess whether FSK increases GluA3 levels on the cell surface of spines, we performed time-lapse two-photon imaging of PCs in cultured organotypic cerebellar slices infected with Sindbis virus to acutely express GluA3 subunits fused to superecliptic pHluorin (SEP). SEP is a pH-sensitive variant of GFP that shows a reduction in fluorescence upon rapid application of acidic (pH 5) ACSF (Figure S4F; Makino and Malinow, 2009). To test whether GluA3 trafficking can be detected with this method, we first triggered LTD chemically by adding the metabotropic mGluR1 receptor agonist DHPG to induce internalization of AMPARs (Linden, 2001). Indeed, application of DHPG to WT PCs expressing SEP-GluA3 produced a significant decrease in SEP fluorescence at spines (p < 0.0001; Figure 4A) and in synaptic strength (p = 0.004 for amplitude and p = 0.04 for frequency; Figure 4B), which is in line with the endocytosis of AMPARs that occurs during the expression of LTD at the PF-PC synapse (Wang and Linden, 2000). In contrast, washing in FSK failed to induce any change in SEP-GluA3 fluorescence at PC spines (0.03% ± 0.015% change, p = 0.4; Figure 4A), even though FSK significantly increased synaptic currents in the SEP-GluA3-expressing PCs (p = 0.04 for amplitude and p < 0.0001 for frequency; Figure 4B). These data suggest that the cAMP-driven synaptic potentiation does not require an insertion of GluA3-containing AMPARs at the surface of spines. To assess whether FSK promotes lateral mobility of GluA3 receptors instead of an increase in receptor externalization, we performed fluorescence recovery after photobleaching (FRAP) experiments of single spines of PCs expressing SEP-GluA3 (Figure 4C). After ∼80% photobleaching, the SEP signals recovered to ∼50%, suggesting that a proportion of SEP-GluA3 is immobilized at synapses (Makino and Malinow 2009). The SEP fluorescence intensity recovered at a similar pace in the presence or absence of FSK (p = 0.9), indicating that the lateral mobility of GluA3-containing AMPARs is not influenced by a rise in cAMP.

**Figure 5:**
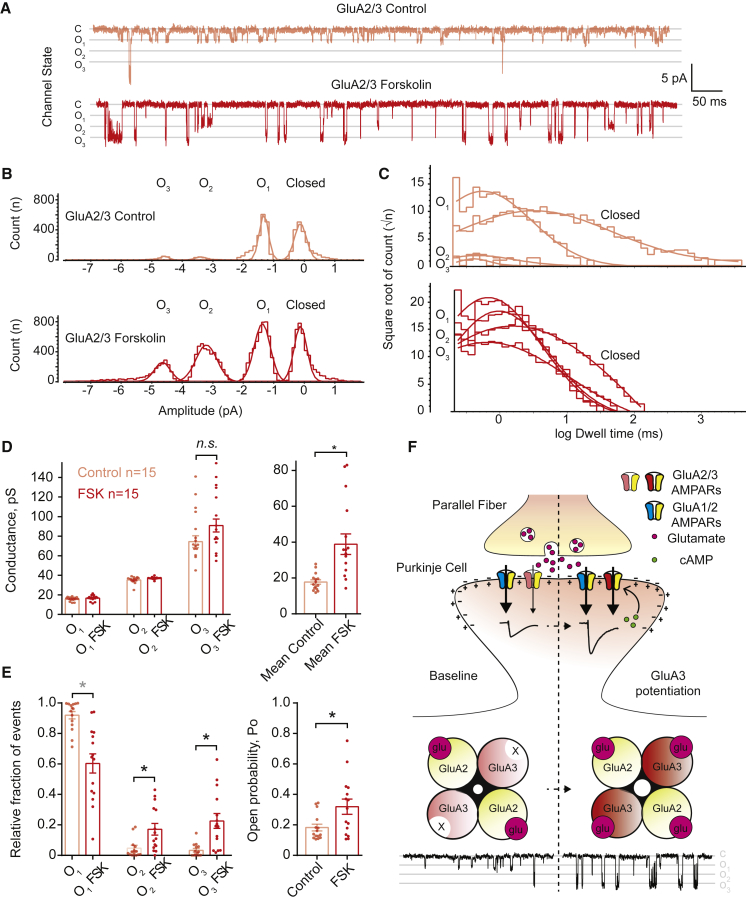
GluA3 Plasticity Occurs through cAMP-Dependent Changes of Single-Channel Conductance and Open Probability (A) Example traces of cell-attached, single-channel recordings of GluA2/GluA3 AMPARs in PCs of GluA1-KO mice. Under basal conditions (light red), the vast majority of the openings of GluA2/GluA3 AMPARs occur at the low conductance level (O1), but in the presence of FSK, the amount of openings in the higher conductance levels (O2-3) increases (red). (B) Count-versus-amplitude histograms of the events detected in the representative recordings shown in (A) illustrate the uneven distribution of events across the different conductance levels in the absence (light red) or presence (red) of FSK. (C) The opening durations (dwell time) of the same events shown in (A) and (B) were unchanged after FSK application. However, the duration of the closed-state times was reduced, suggesting a net increase in the total number of openings produced by GluA2/GluA3 channels in the presence of FSK. (D) Overall quantification of GluA2/GluA3 single-channel recordings shows that the conductance significantly increased in the presence of FSK, yet the conductance per open state remained unchanged. (E) FSK significantly changed the distribution of GluA2/GluA3 AMPAR events, as revealed by a significant decrease of events at O1 state and a significant increase in events at O2 and O3 states. The reduction of the closed-state time shown in (C) was translated into a significant increase of the open-channel probability. (F) The classical model of GluA1-subunit-dependent LTP in pyramidal cells (see Introduction) does not prove valid at PF-PC synapses. Note that the absolute numbers of subsets of both GluA1/GluA2 and GluA2/GluA3 AMPARs are unchanged upon LTP induction, whereas the GluA2/GluA3 AMPARs are activated by cAMP signaling, enhancing their channel conductance and thereby increasing the current generated in potentiated synapses. This model describes for the first time a form of GluA3-dependent LTP. Error bars indicate SEM; ^∗^ indicates p < 0.05.

To assess whether GluA3 plasticity involves a change in channel properties, we resolved single-channel, AMPA-mediated currents by clamping a single AMPAR in cell-attached mode at the cell body of either GluA1-KO or GluA3-KO PCs with the recording pipette containing near-saturating concentrations of AMPA (Poon et al., 2010, Poon et al., 2011). GluA1-containing AMPARs at the surface of GluA3-KO PC cell bodies stochastically reached open states 1, 2, and 3 (indicating binding of 2, 3, and 4 glutamates per receptor complex, respectively) and displayed similar conductance levels and open-channel probability in the presence or absence of FSK application (Figure S5). In contrast, GluA3-containing AMPARs on cell bodies of GluA1-KO PCs produced the vast majority of their openings in the first and lower conductance state (O1) under basal conditions (Figures 5A and 5B), indicating that only two out of the four ligand-binding domains (LBDs) present in the AMPAR tetramer are activated by AMPA. After application of FSK, the behavior of GluA3-containing AMPARs changed strikingly and produced a significantly higher amount of openings in state O2 and O3—similar to GluA1-containing AMPARs (compare Figures 5A, 5B, 5E, and S5B). The average duration of the openings was unchanged (p = 0.4, p = 0.13, and p = 0.09 for O1, O2, and O3, respectively; Figure 5C), but an increase of the absolute frequency of the openings caused shortening of the closed-state dwell-time and thus a significant net increase in open probability (p < 0.0001; Figure 5E). Although FSK did not significantly change the conductance level of any of the open states (p = 0.7, p = 0.14, and p = 0.15 for O1, O2, and O3, respectively; Figure 5D), the higher relative fraction of events in the highly conductive open states O2 and O3 caused a significant increase (p < 0.0001) in the overall conductance of cAMP-stimulated GluA3 channels (Figures 5B and 5D). These experiments suggest that a rise in intracellular cAMP produces synaptic potentiation by increasing the open-channel probability of the GluA3 subunit, indicating a novel mechanistic model for GluA3-dependent synaptic plasticity.

### GluA3-Mediated Plasticity Is Induced via cAMP-Mediated Epac Activation

**Figure 6:**
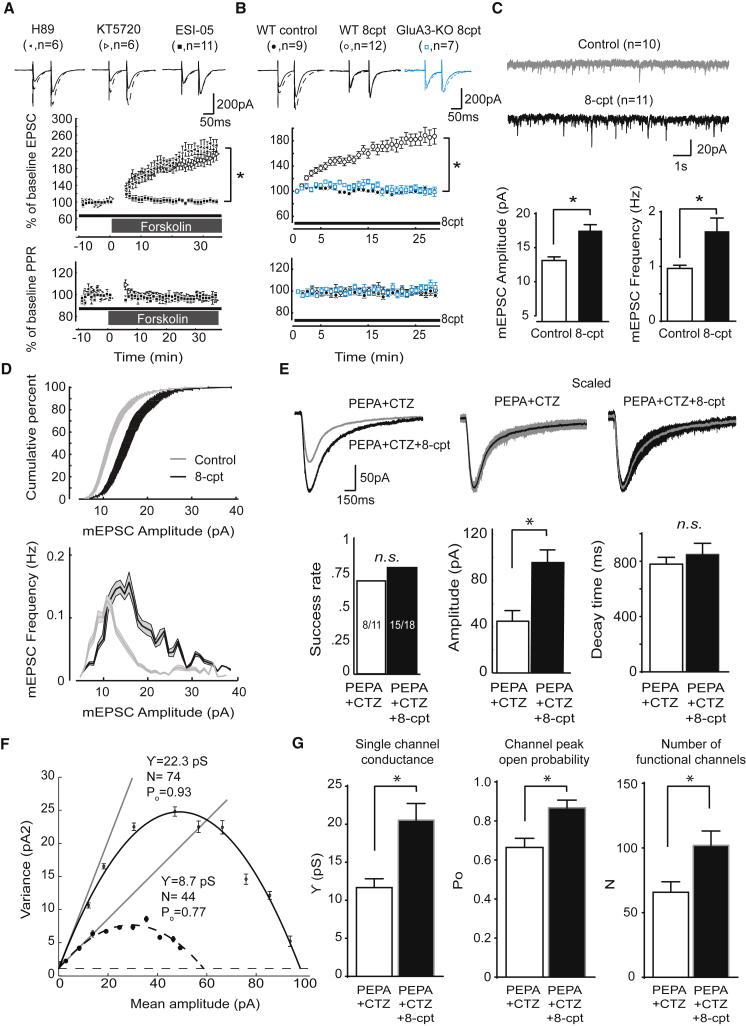
GluA3 Plasticity Requires cAMP-Dependent Postsynaptic Activation of Epac (A) Epac2 antagonist ESI-05 blocks FSK-driven synaptic potentiation, whereas PKA antagonists H89 and KT5720 do not. (B) Intracellular application of membrane-impermeable Epac agonist 8CPT caused significant synaptic potentiation in WT PCs (open circles) compared with GluA3-KO PCs (blue boxes) or the no-drug condition in WT PCs (closed circles). (C) Intracellular application of 8CPT caused an increase in both mEPSC amplitude (left) and frequency (right). (D) A shift toward higher mEPSC amplitudes was visualized both in the cumulative distribution and in the mEPSC frequency-versus-amplitude distribution plots, once again suggesting postsynaptic effects of EPAC activation. (E) Outside-out patches excised from PC somata recorded in the presence of AMPAR desensitization blockers (PEPA and CTZ) had a similar success rate of containing AMPA events (left), but generated significantly larger currents (middle) with similar decay time kinetics (right) when 8CPT was present in the internal solution. (F) Example parabolic distribution of the variance-versus-amplitude relationship obtained from bins of the current decay profile. Nonstationary noise analysis (NSNA) was done by fitting a parabolic equation to this distribution in order to estimate conductance, open probability, and number of active receptors. (G) NSNA performed on the PC recordings in (E) revealed significantly increased single-channel conductance (left) and peak open-channel probability upon 8CPT application (middle), which in turn led to an increased number of functional channels responding to the local AMPA application (right). Error bars indicate SEM; ^∗^ indicates p < 0.05.

**Figure 7:**
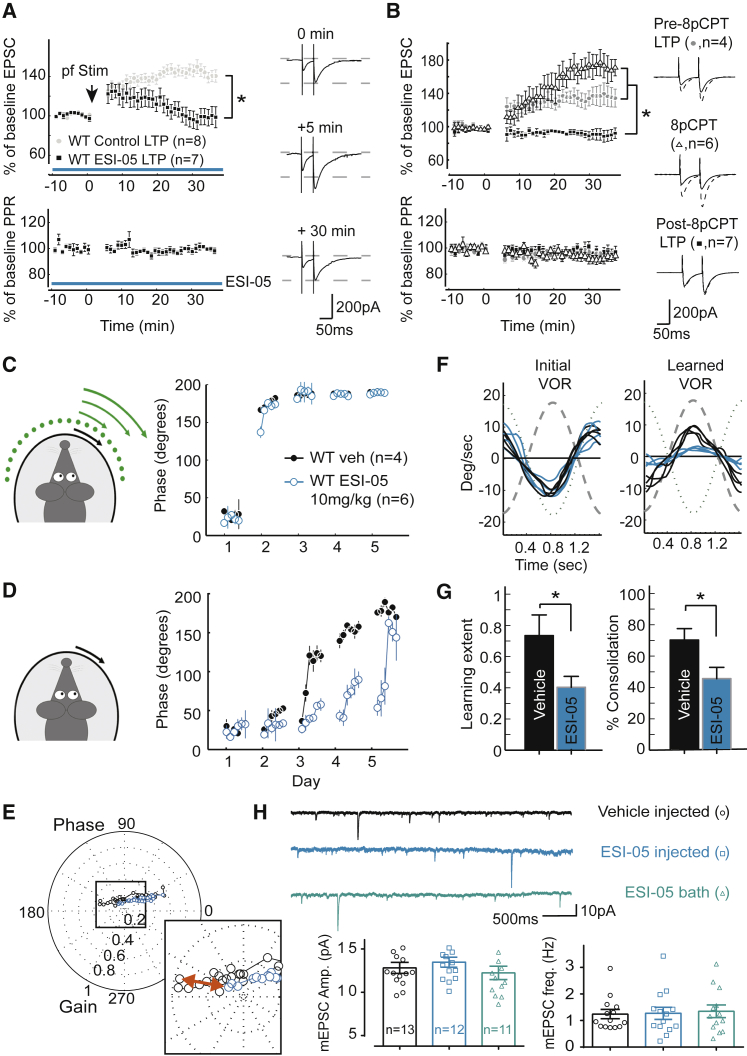
Pharmacological Manipulation of Epac Activity Impairs LTP In Vitro and Motor Learning In Vivo without Affecting Synaptic Transmission (A) Epac antagonist ESI-05 prevents PF-PC LTP induced by tetanic PF stimuli. (B) Epac activation through incubation with 8pCPT potentiates AMPAR currents (open triangles). As a consequence, a minimal 30 min incubation with 8cCPT fully occludes LTP induction (black squares) compared with LTP induction in the absence of 8cCPT (gray circles). (C) Eye-movement phase values in WT mice that are injected with 10 mg/kg ESI-05 (open blue circles) or with vehicle only (black circles) are virtually identical during visuovestibular mismatch training when the light is on. (D) During the catch trials in the dark, the phase shift of VOR adaptation in the mice injected with 10 mg/kg ESI-05 is significantly delayed compared with the phase shift in their littermates injected with vehicle only. (E) Polar plot of the combined gain and phase data shows a common learning trajectory and comparable initial gain, yet a different final outcome, for both groups. In the inset, the final VOR reached after 5 days of training is amplified to visualize the magnitude of the gain difference (red arrow) between ESI-05-injected and vehicle-injected mice. (F) Four representative eye-velocity traces of the VOR before (left) and after (right) phase-reversal training show that, whereas both ESI-05 and vehicle-injected mice show equal baseline performance and both are able to flip the phase of the VOR, the magnitude of the VOR reached after the training is substantially different. (G) Both learning extent and consolidation during the phase-reversal task are significantly smaller in the mice injected with ESI-05 than in those injected with vehicle only (T2 test p < 0.05). (H) Impaired motor learning after ESI-05 injections does not correlate with decreased transmission at PF-PC synapses. The PC mEPSC amplitude and frequency did not change upon injection of WT mice with ESI-05 or upon incubation of WT slices with ESI-05. Error bars indicate SEM; ^∗^ indicates p < 0.05.

To further elucidate the molecular mechanism underlying GluA3-dependent plasticity, we aimed to identify the intermediary factor that translates a rise in cAMP into synaptic potentiation of GluA3-containing AMPARs. Protein kinase A (PKA) is activated by a rise in cAMP and exerts cAMP-dependent synaptic effects (Lev-Ram et al., 2002, Sokolova et al., 2006). However, incubating WT PCs with PKA antagonist KT5720 or PKA antagonist H89 did not have a significant effect on synaptic potentiation induced by FSK (215% ± 20% with KT5720 and 235% ± 19% with H89; p = 0.7 and p = 0.9, respectively; Figure 6A), indicating that PKA is not involved in mediating GluA3 plasticity. We next assessed the involvement of Epac (exchange proteins directly activated by cAMP, a.k.a. Rap guanine-nucleotide-exchange factor) as an alternative cAMP-dependent pathway that can trigger synaptic changes (Gekel and Neher, 2008, Woolfrey et al., 2009). The blockade of Epac with its selective antagonist ESI-05 (Tsalkova et al., 2012) did not reduce basal transmission at PF-PC synapses (Figure 7H), but it effectively prevented the FSK-induced synaptic potentiation in WT PCs (p = 0.9 versus baseline and p < 0.0001 versus control condition without ESI-05) (Figure 6A). To assess whether Epac activation is not only necessary but also sufficient to cause GluA3-dependent synaptic potentiation, we investigated the impact of the selective Epac activator 8-CPT-2Me-cAMP (8CPT). Adding 20 μM 8CPT to the intracellular recording solution produced synaptic potentiation in WT PCs (185% ± 17%, p = 0.0004; Figure 6B), but not in GluA3-deficient PCs (100% ± 5%, p = 0.8; Figure 6B). In addition, the postsynaptic application of 8CPT increased the amplitude and frequency of PC mEPSCs (p = 0.0005 and p = 0.001, respectively; Figures 6C and 6D) and did not change the PPF ratio (104% ± 5%; Figure 6B). These experiments indicate that a rise in cAMP triggers synaptic potentiation through Epac-mediated activation of postsynaptic, GluA3-containing AMPARs. This Epac-driven activation of GluA3-containing AMPARs was not limited to AMPARs located at synapses. Outside-out patches excised from WT PC somata produced a peak current of approximately 10 pA in response to puffs of 100 μM AMPA (Figure S4E). When the Epac activator 8CPT was added to the internal solution of the patch pipette, the peak current obtained under the same conditions was increased 2.5-fold in the absence of a presynaptic component (25 ± 3 pA, p < 0.0001 versus control). This difference in peak current was largely maintained in the presence of AMPAR-desensitization blockers PEPA and cyclothiazide (45 ± 8 pA without 8CPT versus 97 ± 10 pA with 8CPT, p < 0.0001; Figure 6E), indicating that cAMP-driven GluA3 plasticity does not depend on a change in the desensitization properties of AMPAR channels. As expected from our single-channel results, nonstationary noise analysis of these nondesensitizing AMPAR responses showed a significant increase in conductance and open probability (Figures 6F and 6G). This analysis revealed how, in a mixed pool of GluA1- and GluA3-containing AMPARs, Epac-dependent GluA3 potentiation was translated into an increase in current amplitude without altering the dynamics of the response (Figure 6E), highlighting the consistent results with miniature and evoked EPSC recordings.

### Epac Activation Is Required for LTP and Motor Learning

We next tested whether PF-PC LTP depends on Epac activation. Incubation of slices with Epac inhibitor ESI-05 significantly inhibited synaptic potentiation induced by tetanic PF stimulation (102% ± 13% versus 140% ± 8% in control conditions, p < 0.0001 after 15 min; Figure 7A). In addition, LTP was fully occluded when brain slices were preincubated with the membrane-permeable analog of the Epac activator (8pCPT) (GluA3-KO versus WT, p = 0.008; Figure 7B). Together, these data indicate that Epac2 activation is responsible for postsynaptic LTP at the PF-PC synapse through activation of GluA3-containing AMPARs.

To investigate the involvement of Epac activation in cerebellar synaptic plasticity in vivo, we performed phase-reversal adaptation in WT mice that received daily IP injections either with Epac antagonist ESI-05 (0.2–0.3 mL at 10 mg/kg) or with vehicle alone 30 min prior to the training protocol. Mice administered ESI-05 had unaffected basal-eye-reflex behavior, but performed significantly worse in the phase-reversal task than did vehicle-injected animals (Figure 7D). Although both groups eventually reached a reversal of the VOR phase (Veh, 157% ± 2%, and ESI-05, 148% ± 15%; Figure 7D), its magnitude was significantly lower in the ESI-05-injected mice than in vehicle controls (p = 0.01; Figures 7E–7G). This difference reached after training could not be explained by a poor basic response to the training stimuli (Figure 7C), but only by a significantly reduced learning extent (p = 0.01) and consolidation rate (p = 0.03). Importantly, systemic ESI-05 injections produced learning deficits without a change in basal synaptic transmission compared to vehicle-injected mice (p = 0.5 and p = 0.9 for mEPSC amplitude and frequency, respectively; Figure 7H), suggesting that absence of Epac-dependent synaptic potentiation without any change in basal transmission is sufficient to impair learning capabilities.

### GluA3 Expression in PCs Is Required for VOR Learning

**Figure 8:**
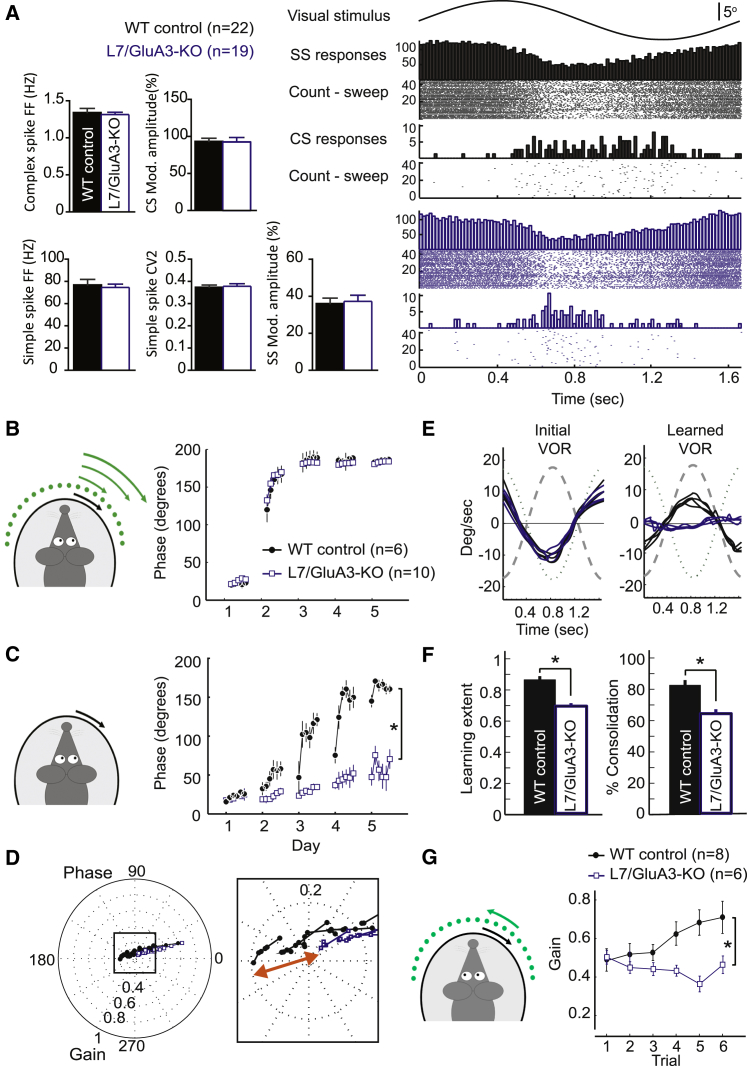
Lack of GluA3 in PCs Causes Motor Learning Deficits (A) Representative activity of vertical-axis PCs recorded in the flocculus of WT and L7/GluA3-KO mice during visual stimulation (5°, 0.6 Hz). Bar graphs show that the averages of firing frequency (FF), the coefficient of variation in adjacent intervals (CV2), the modulation amplitude of simple spikes, and the frequency and modulation amplitude of complex spikes during OKR stimulation were similar in control (n = 22) and L7/GluA3-KO mice (n = 19). The visual stimulus is shown together with histograms of simple spike and complex spike frequencies and corresponding raster plots on the right. (B) Eye-movement phase values in L7/GluA3-KO mice (open square) and WT mice (closed circle) during visuovestibular mismatch training are comparable, highlighting that the strength of the visual signals was in principle sufficient to induce learning. (C) Phase values of VOR-adaptation catch trials in L7/GluA3-KO mice show a significantly impaired shift over 5 days compared with trials in their WT littermates, illustrating that motor learning is affected despite normal visual signaling as demonstrated in (A) and (B). (D) Polar plot of the gain and phase data shows a common learning trajectory and comparable initial gain for both groups. In the inset, the final VOR reached after 5 days of training is amplified to visualize the magnitude (red arrow) of the gain difference between L7/GluA3 KO and WT mice. (E) L7/GluA3-KO mice (blue line) show equal baseline performance to WT mice (black line), but are unable to reverse the phase of their VOR. Data show four representative eye-velocity traces of the VOR before (left) and after (right) phase-reversal training. (F) Both learning extent and consolidation during the phase-reversal task are significantly smaller in L7-GluA3 KO mice than those in WT littermates (T2 test, p < 0.05). (G) Gain-increase learning reveals deficits for L7/GluA3-KO mice compared to WT mice. Error bars indicate SEM; ^∗^ indicates p < 0.05.

We showed that VOR learning depends on global expression of GluA3 and that LTP at PF-PC synapses requires GluA3 plasticity—but does VOR learning depend on GluA3 specifically in PCs? To address this question, we generated and tested a PC-specific GluA3-KO mouse (referred to as L7/GluA3-KO; Figure S6) by crossbreeding mice expressing Cre-recombinase under the PC-specific promoter L7-pcp2 with mice in which the GluA3 gene is flanked by loxP sites (Barski et al., 2000, Sanchis-Segura et al., 2006). After establishing the single-unit identity of floccular vertical-axis PCs in adult L7/GluA3-KO mice by demonstrating a CF pause in their simple spike activity as well as a preferred modulation tuning-curve during extracellular recordings in vivo (Figure 8A), we investigated the action-potential generation of their simple spike activity in the awake state. In the absence of visual stimulation, both the firing frequency and regularity (i.e., coefficient of variation of adjacent interspike intervals, or CV2) of the simple spike activity of the L7/GluA3-KO mice did not differ significantly from those of WTs (Figure S7A), which is consistent with the similar I-V relationships recorded in vitro for WT and GluA3-lacking PCs (Figure S7B). Next, we provided visual stimulation at the frequency that was used for the training paradigm (0.6 Hz). Again, the firing frequency and regularity (i.e., CV2) of the simple spike activity of the L7/GluA3-KO mice did not differ from those of WTs (p = 0.7 and p = 0.8, respectively; Figure 8A). Moreover, and most importantly, the amplitude of the simple spike modulation during visual stimulation did not differ (p = 0.8; Figure 8A), suggesting that the PF output is, in effect, sufficient to mediate the visual training signals in the L7/GluA3-KO mice despite the reduced PF-PC synaptic transmission (Figure S7C). Finally, the firing frequency and modulation amplitude of the complex spikes did not show any significant difference, either (p = 0.7 and p = 0.9, respectively; Figure 8A). Together, these data indicate that the in vivo excitability and spike generation of PCs are intact in L7/GluA3-KO mice.

We then tested 3- to 5-month-old L7/GluA3-KO and control littermates for their ability to adapt eye reflexes. The baseline OKR and VOR performances of these L7/GluA3-KO mice were indistinguishable from those of controls (Figure S1). Similarly to global GluA3-KOs, VOR motor learning was prominently affected in L7-GluA3-KO mice (Figures 8C–8G). Mice lacking GluA3 in PCs showed significant deficits throughout the phase-reversal paradigm (all p values < 0.01 after day 2) and had a significantly different learning extent at the end of the paradigm (p = 0.0005; Figures 8B–8F). Moreover, consolidation during the phase-reversal paradigm was significantly lower (p = 0.0006, Figure 8F). Gain modulation was also impaired, as shown by a significant difference between the final eye movement gain of L7/GluA3-KO mice and that of their control littermates after either gain-down or gain-up training sessions (p = 0.04 and p = 0.006 for gain-down and gain-up, respectively) (Figure 8G).

In contrast to the single L7/GluA3-KO as well as the single global GluA1-KO and GluA3-KO mice, mice that lacked both GluA1 and GluA3 receptor subunits specifically in PCs (L7-GluA1/GluA3-KO) showed significant aberrations in baseline eye movement performance (p = 0.0001 for OKR and p = 0.03 for VORD; Figures S1A and S1B). Together with the findings presented above (see also Figure 1), these data suggest that the presence of GluA3 in the GluA1-KO mouse can compensate for its lack of GluA1 during both baseline and learning behavior, but the presence of GluA1 in the global GluA3-KO mouse and single L7-GluA3-KO mouse is not sufficient to fully compensate for the lack of GluA3 during adaptation of compensatory eye movements. This highlights the putative impact of GluA3-dependent synaptic plasticity in PCs.

## Discussion

It is widely believed that LTP- and LTD-type synaptic plasticity mechanisms act in concert to mediate several types of learning in brain regions such as the hippocampus, amygdala, and cerebral cortex (Malinow and Malenka, 2002, Makino and Malinow, 2011, Nabavi et al., 2014, Nedelescu et al., 2010, Rumpel et al., 2005, Takahashi et al., 2003). For cerebellar learning, LTD at the PF-PC synapse has historically been considered the dominant plasticity mechanism (Ito, 2002, Linden and Connor, 1995). Although the simple spike suppression observed at early stages of some forms of motor learning in vivo may suggest LTD occurrence (ten Brinke et al., 2015, Yang and Lisberger, 2014), an increasing number of studies suggest that LTD is not a strict requisite for motor learning (Hesslow et al., 2013, Schonewille et al., 2011). In the present study, we show that LTP at PF-PC synapses is a required mechanism for cerebellar motor learning. We show that LTP, but not LTD, at the PF-PC synapse requires plasticity of GluA3-containing AMPARs and that both the selective removal of GluA3 in PCs and the pharmacological blockade of the pathway leading to GluA3 plasticity in vivo severely impair the ability to adapt the vestibulo-ocular reflex. Combined, these findings provide the first correlative link between GluA3-dependent LTP and behavioral learning in general.

Previous studies proposed a role for cerebellar LTP in the context of bidirectional gain modulation (Boyden et al., 2006). This work suggested that gain-down modulation of eye movements might require PF-PC LTP and, conversely, that gain-up modulation would require LTD. According to this hypothesis, one would expect GluA3-KO mice to show impaired gain-down modulation with intact gain-up adaptation. However, our data show that the specific absence of GluA3 in PCs most prominently impairs gain-up and phase modulation, supporting an opposite kind of role for GluA3-dependent LTP in oculomotor learning. Whereas the role of GluA3 in PC plasticity and cerebellar motor learning is becoming more clear now, the role of GluA1 is still largely obscure. The presence of GluA1 in PCs was essential neither for the induction of LTD nor of LTP, and there were no overt signs of deficits in motor performance or motor learning in the GluA1-KO mice. Its possible role became only indirectly apparent, when we observed that, in contrast to the single GluA1-KO mice, the double GluA1/GluA3-KO mice virtually completely lacked glutamatergic currents in PCs, and the double L7-GluA1/GluA3-KO mice showed significant signs of ataxia and deficits in motor learning. Given that the single GluA3-KO mice did not show any sign of motor performance deficit, these findings indicate that GluA1-containing AMPARs in PCs do contribute to cerebellar motor performance but that their absence can be compensated for by GluA3-containing AMPARs.

The possible role of LTP at the PF-PC synapse in cerebellar motor learning has been suggested before by various other cell-specific mouse mutant studies (Andreescu et al., 2007, Schonewille et al., 2010, Peter et al., 2016). However, these studies tackled more upstream PC processes that involved the nuclear estrogen receptor, cytosolic protein phosphatase calcineurin, and subsynaptic protein shank2, and as a consequence they suffered from various side effects that prevented definitive conclusions (Gao et al., 2012). In the current study, in which we tackled PF-PC LTP more downstream by targeting GluA3-containing AMPARs at the level of the synapse itself, we did not find any evidence for structural changes or firing differences in PCs of awake behaving mice. We did find that the basal transmission was reduced in PCs lacking GluA3 (both in the global and the cell-specific KO mice), but this deficit was probably not the reason for the impairment of LTP or of motor learning because acutely inhibiting GluA3 plasticity through blockade of Epac prevented both LTP and motor learning without affecting basal transmission. We therefore propose that the reduced basal transmission in GluA3-KO mice is the consequence of a prolonged deficit in LTP.

GluA1-dependent synaptic plasticity is mediated by active trafficking (Makino and Malinow, 2011, Shi et al., 2001) and by changes in conductance and open probability at the single receptor level (Benke et al., 1998, Derkach et al., 1999). Here we present evidence that, at least at the short-term scale of tens of minutes, synaptic potentiation through activation of GluA3 plasticity does not involve trafficking but mainly involves a prominent increase in open-channel probability of GluA3-containing receptors, suggesting that in the case of GluA3, a change in receptor properties is the predominant mechanism for producing synaptic potentiation. These findings imply that PF-PC LTP is, mechanistically, not just the reverse of LTD at this synapse (Jörntell and Hansel, 2006). Linden and colleagues have shown that PF-PC LTD is largely expressed by endocytosis of GluA2-containing AMPARs (Linden, 2001, Schonewille et al., 2011) and thus mainly dependent on AMPAR trafficking. Given the current findings on GluA3-mediated LTP, it may be worthwhile to find out whether changes in AMPAR unitary conductance or glutamate affinity also play a minor role in early LTD expression at the PF-PC synapse, as interference with clathrin-mediated endocytosis did not produce a total attenuation of LTD expression (Wang and Linden, 2000).

Full genetic ablation of GluA2 subunits, in contrast to that of GluA3, produces an ataxic, hardly viable phenotype (Gerlai et al., 1998). Interestingly, the remaining mainly GluA1- and GluA3-containing AMPARs in these KO mice have an unusual subunit composition and are abnormally distributed at the synapse (Sans et al., 2003). In this respect, it should be noted that GluA3 is an obligatory heteromeric subunit: GluA3 homomers are energetically unfavorable (Rossmann et al., 2011) and form intracellular aggregates that do not reach the cellular surface efficiently (Coleman et al., 2016). Genetic mouse models in which GluA2 trafficking is blocked reveal an impairment in LTD induction at their PF-PC synapses, whereas LTP is normal (Schonewille et al., 2011, Steinberg et al., 2006), which is in line with our finding that LTP can be induced without trafficking of GluA2/GluA3-containing AMPARs. Differently from these GluA2 mutants, GluA3-KO mice prominently express surface GluA2-containing AMPARs (heteromerized with GluA1) but lack a cAMP-dependent synaptic LTP. These data highlight the differential roles of GluA2 and GluA3 in the structural dynamics and localization of AMPARs and the related forms of synaptic plasticity. In contrast to GluA3, GluA2 is unlikely to be directly involved in cAMP-dependent plasticity, since its expression coupled to GluA1 does not compensate for the absence of GluA3 subunits. We propose that GluA2 expression is a structural requisite for GluA3 plasticity, as it appears necessary for proper expression and location of GluA3-containing AMPARs. GluA1/GluA2 heteromers in PCs may then serve to maintain basal synaptic currents when cAMP levels are low.

The finding that an Epac-mediated change in single-channel conductance and open probability of GluA2/GluA3-containing AMPARs may underlie LTP at the PF-PC synapse raises the question of how this change in configuration comes about. Interestingly, the distribution of GluA3-containing AMPARs openings does not seem to respond to a stochastic probability distribution of four LBDs “catching” glutamate with equal probability. Instead, it is biased toward the lowest conductance-state opening, in which only two out of four LBDs bind glutamate. Since GluA3-containing AMPARs predominantly consist of two GluA3 and two GluA2 subunits, only the GluA2 LBDs may effectively bind glutamate under basal conditions. Our observation that enhancing cAMP levels exerts GluA3-containing receptors to produce higher conductance openings (resembling the behavior of GluA1-containing receptors) may suggest that Epac activation triggers a conformational change in the two GluA3 subunits present in each tetramer, such that they become responsive to glutamate binding at the LBD (Figure 5F; Sukumaran et al. 2011).

It is widely accepted that intracellular calcium signaling is a key mechanism for LTP induction in PCs (Coesmans et al., 2004, van Woerden et al., 2009). In the present study we show that postsynaptic LTP depends on cAMP-dependent activation of GluA3-containing receptors. How low calcium signals in PCs are transduced into activation of adenylyl cyclase to raise cAMP levels remains to be elucidated. Interestingly, it has been shown that the tetanic activity of PFs required for LTP induction produces local calcium increases dependent on low-threshold CaV3.1 T-type calcium channels (Hildebrand et al., 2009) and that global deletion or blockage of these channels prevents LTP induction and motor learning (Ly et al., 2013). In this respect, the calcium/calmodulin-dependent adenylyl cyclase Adcy1 (Masada et al., 2012) could be an interesting candidate to convert a local calcium signal into a rise in cAMP.

We have shown here that postsynaptic, GluA3-dependent synaptic potentiation depends on a rise in cAMP. Therefore, this study expands the repertoire of forms of PC plasticity already known to depend on cAMP, such as presynaptic plasticity (Chen and Regehr, 1997, Kaneko and Takahashi, 2004, Lev-Ram et al., 2002, Salin et al., 1996), intrinsic plasticity (Belmeguenai et al., 2010), or plasticity at inhibitory synapses (Mitoma and Konishi, 1996). Epac2 has recently been reported to also have a role in presynaptic plasticity, in that it may modify glutamate release probability (Gekel and Neher, 2008). This raises the interesting possibility that Epac2 and/or cAMP, in their presynaptic and postsynaptic domains, operate in a synergistic fashion to control synaptic plasticity (Le Guen and De Zeeuw, 2010, Wang et al., 2014). Likewise, the induction protocol of LTP produces an increase in intrinsic excitability in PCs via cAMP-mediated PKA modulation of SK potassium channels (Belmeguenai et al., 2010). Thus, since this change in intrinsic excitability occurs at least partly as a secondary process following tetanic PF stimulation, LTP at the PF-PC synapse may act as a feedforward amplifier of synaptic inputs to modulate firing rate in PCs via cAMP production. Finally, it should be noted that rebound potentiation at the molecular-layer interneuron-to-PC synapse, which occurs following PC depolarization, is also mediated by cAMP-mediated PKA modulation (Hirano and Kawaguchi, 2012). Together, these findings point toward a central role of cAMP following induction of PF-PC LTP in regulating multiple forms of plasticity with different identities and natures in a synergistic fashion (Gao et al., 2012).

Synapses are highly dynamic structures, and early removal of synaptic proteins can lead compensatory mechanisms to occur in order to overcome unbalanced synaptic function. However, no compensatory mechanism is able to overcome the declarative memory deficits observed in GluA1-KO mice (Feyder et al., 2007, Humeau et al., 2007). In contrast, GluA1-KO mice learned to adapt their vestibulo-ocular reflexes virtually identically to WT littermates. This finding suggests two possible scenarios: either PC synapses are capable of compensating for the absence of GluA1 through a mechanism that is not present in hippocampal pyramidal or amygdalar cells, or GluA1 is not involved in this form of learning at all. With the evidence presented here, neither of these possibilities can be unequivocally discarded. Yet these findings in GluA1-KO mice emphasize the insufficiency of compensation in GluA3-KO mice; the fact that their PCs could not compensate for the absence of GluA3 to overcome the lack of LTP and the learning deficits highlights the importance of GluA3 for PC synaptic plasticity and motor learning. Taken together, the picture emerges that the learning rules for AMPAR-mediated plasticity in PCs are inverted compared with those in the hippocampus: cerebellar LTP and learning do not require GluA1 but depend on the plasticity of GluA3-containing AMPARs.

## Experimental Procedures

Below, the experimental methods are briefly summarized; for extended experimental procedures, which have all been done in a blinded fashion, see Supplemental Information.

### Mice

GluA1-KO mice were generated by mating heterozygous c57bl6/129 mice (Kim et al., 2005), GluA3-KO mice were bred from c57bl6x129P2-Gria3tm1Dgen/Mmnc mutant ancestors (MMRRC), and PC-specific GluA3-KO mice were generated by crossing floxed GluA3 mice (Sanchis-Segura et al., 2006) with L7-Cre mice (Barski et al., 2000).

### Eye Movement Recordings

Mice were prepared for chronic experiments (de Jeu and De Zeeuw, 2012). Eye orientation was measured using video pupil tracking (Pulnix TM-6710CL). Online image analysis was performed using custom-built software (National Instruments). Angular eye velocity was computed offline (Stahl et al., 2000). The horizontal VOR was characterized using sinusoidal rotation about the vertical axis and subsequently subjected to a VOR cancellation and reversal stimulus.

### In Vitro Electrophysiology

Sagittal slices of the vermis were obtained in ice-cold “slicing” solution, and subsequently transferred to the same solution at 34°C. Whole-cell patch-clamp recordings were performed using an EPC-10 amplifier (HEKA, Lambrecht). PF-PC LTD was induced by pairing PF stimulation with somatic depolarization (Linden, 2001) or CF stimulation (Schonewille et al., 2011). PF-PC LTP was induced by PF stimulation alone (Schonewille et al., 2010). Cells with more than a 20% change in series resistance over time after plasticity induction were discarded for analysis (Figure S8). Single-channel activity was measured in cell-attached configuration. The driving potential, resulting from subtraction of the resting potential and clamped voltage, was used to calculate the receptor conductance. For the outside-out patches, pipettes with 4–6 MΩ resistance were used to establish Giga-seals. After breaking into whole cell mode, the pipette was retracted until both the cell and the outside-out patch were re-sealed. Spontaneous mEPSC and evoked EPSC recordings were analyzed with MiniAnalysis software (Synaptosoft) and ClampFit (Molecular Devices). The decay time constant for AMPA-evoked currents in outside-out patches in the presence of desensitization blockers was calculated by dividing the total charge transfer (in fC) by the peak amplitude (in pA). Nonstationary fluctuation analysis of outside-out patch traces was carried out according to Hartveit and Veruki (2007).

### Drugs

For mEPSC recordings, TTX (Sigma) was added to the bath solution to only measure excitatory spontaneous release. For investigation of the cAMP-GluA3-dependent pathway, we used FSK (Sigma), H89 (Tocris), KT5720 (Sigma), ESI-05 (BioLog), and 8-CPT-2Me-cAMP (Tocris Bioscience). To obtain a monophasic time decay of the AMPA-evoked responses in outside-out patches, we added PEPA (Tocris bioscience) and cyclothiazide (Tocris bioscience).

### In Vitro Two-Photon Imaging

Organotypic cerebellar slices were transfected with sindbis-virus-expressing rat GluA3(i) fused to the pH-sensitive version of GFP super-ecliptic pHluorin (SEP-GluA3). For imaging, slices were transferred from the incubation solution to the recording chamber containing ACSF. Three-dimensional images were collected, and optical sections were captured from transfected PC dendrites using ImageJ software (NIH). For single-spine bleaching in the FRAP experiments, a ROI was selected covering the surface of a single spine.

### In Vivo Electrophysiology

Mice were prepared for chronic experiments (Schonewille et al., 2010). A recording chamber was constructed around a small craniotomy, and animals were habituated in the setup. Extracellular activities were recorded with glass micropipettes filled with 2M NaCl solution and advanced into the cerebellar cortex. Electrode signals were stored for offline analyses (Spike2, CED, and Cambridge, UK). PCs were identified by the occurrence of both simple spikes and complex spikes, and single-unit activity was confirmed by a brief pause in simple-spike firing following each complex spike. The whole-field visual stimulation was presented by rotating a cylindrical screen. Offline analysis was conducted in MATLAB (Mathworks).

### Statistics

For statistical analysis, we used either MATLAB statistical toolbox (MathWorks) or GraphPad Prism 6.

## Author Contributions

N.G.-C., C.M.D.S.-M., O.O., H.W.K., and C.I.D.Z. designed, performed, and analyzed the behavioral and histological experiments; N.G.-C., L.M.C.K., K.Z., C.B.C., M.C.R., H.W.K., and C.I.D.Z. designed, performed, and analyzed the electrophysiological and pharmacological experiments; R.S. generated conditional AMPAR-KO mice; and N.G.-C., C.B.C., H.W.K., and C.I.D.Z. wrote the manuscript. H.W.K. and C.I.D.Z. contributed equally to this work.

## Supplemental Information

Document S1. Supplemental Experimental Procedures and Figures S1–S8

Document S2. Article plus Supplemental Information
