# Supplementary material for: Motor Learning Requires Purkinje Cell Synaptic Potentiation through Activation of AMPA-Receptor Subunit GluA3
Source: Neuron. 2017 Jan 18;93(2):409–24. doi: 10.1016/j.neuron.2016.11.046 (PMC5263704; doi:10.1016/j.neuron.2016.11.046)
Supplement: Document S1. Supplemental Experimental Procedures and Figures S1–S8 [file mmc1.pdf]

Neuron, Volume *93*

Supplemental Information

Motor Learning Requires Purkinje Cell Synaptic Potentiation through Activation of AMPA-Receptor Subunit GluA3

Nicolas Gutierrez-Castellanos, Carla M. Da Silva-Matos, Kuikui Zhou, Cathrin B. Canto, Maria C. Renner, Linda M.C. Koene, Ozgecan Ozyildirim, Rolf Sprengel, Helmut W. Kessels, and Chris I. De Zeeuw

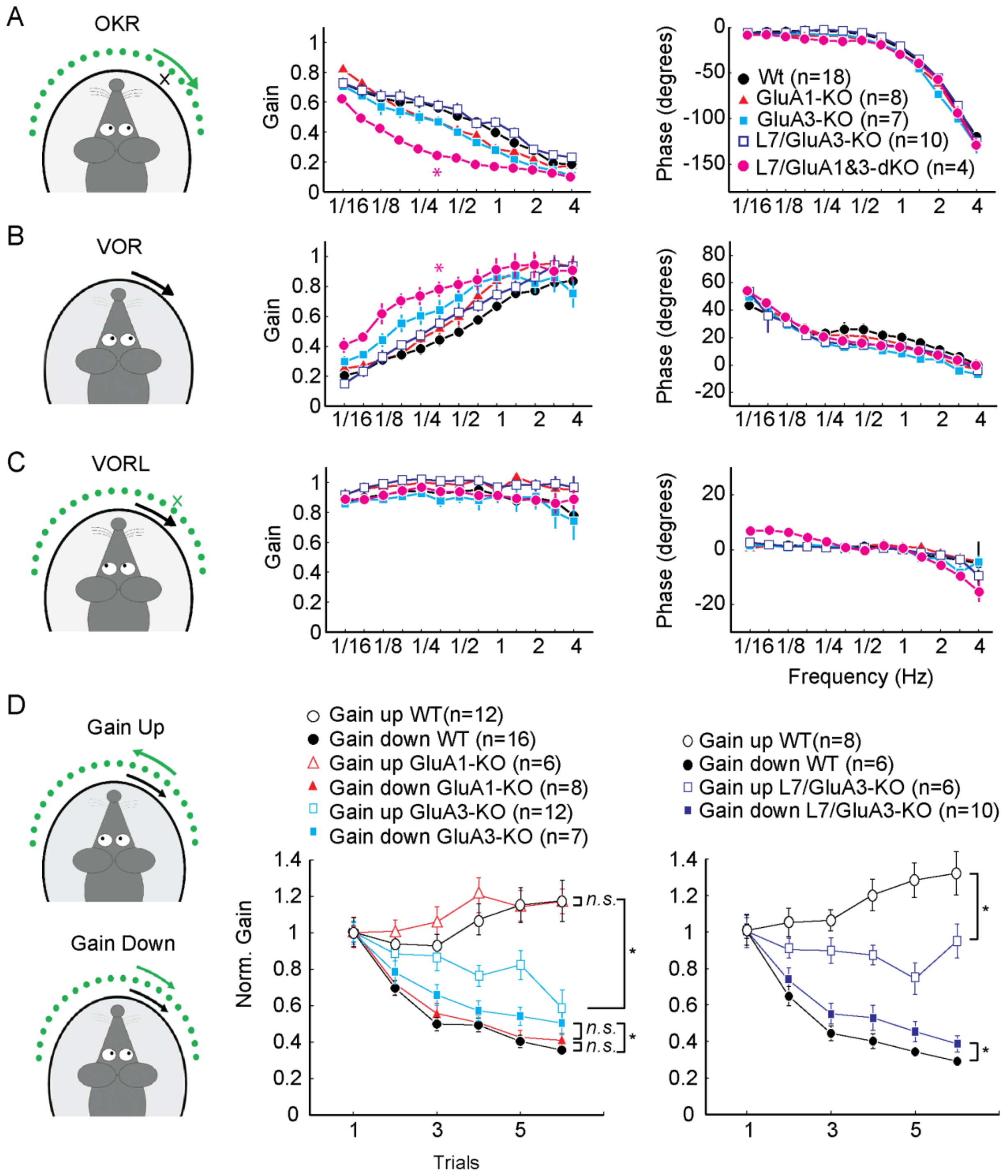

Figure S1. Related to Figures 1 and 8, Basic compensatory eye movements in the single global KO mice for GluA1 or GluA3 as well as in PC-specific KO for GluA3 are relatively normal, whereas those in the double PC-specific KO for GluA1 and GluA3 show pronounced gain deficits.

(A) The optokinetic reflex (OKR), which stabilizes gaze with respect to a moving visual field (Stahl et al., 2000), showed a normal baseline gain in GluA1-KO and GluA3-KO mice ($F(2,29)=2.361$, $p = 0.11$; Repeated measures ANOVA and Tukey post hoc analysis), whereas phase values in both mutants presented a small, but consistent, delay across the entire frequency range tested ($F(2,29)=14.86$, $p <$

0.01, Tukey's multiple comparisons test revealed differences for the 95% confidence intervals of both mutants with respect to wild-type controls, but not between them). L7/GluA3-KO mice presented intact gain and phase values compared to controls ($F(1,26) = 0.21$, $p = 0.64$ and $F(1,26) = 1.24$, $p = 0.27$, respectively). In contrast, PC-specific double GluA1 and GluA3 KO mice (L7/GluA1&3-dKO) showed a highly significantly impaired OKR response ($F(1,20) = 21.30$, $p = 0.0001$)

(B) During VOR compensation GluA1-KO and GluA3-KO showed both a normal gain ($F(2,29)=1.745$, $p = 0.17$) and normal phase ($F(2,29)=1.382$, $p = 0.26$). In addition, L7/GluA3-KO mice also showed a normal basic eye movement performance ($F(1,26) = 1.65$, $p = 0.21$ and $F(1,26) = 1.53$, $p = 0.22$, for gain and phase, respectively). Interestingly, L7/GluA1&3-dKO showed significantly improved VOR performance when compared to control mice ($F(1,20) = 5.245$, $p= 0.033$), most likely as a compensation for their impaired OKR.

(C) When we combined optokinetic stimulation with vestibular stimulation (i.e. VOR in the light or VORL) as occurs in daily life, all mutants also showed normal performances for both gain and phase compared to those in wild-type littermates ($F(2,29) = 1.33$, $p = 0.29$ for GluA1-KO, GluA3-KO and their WT littermates and $F(1,26) = 1.51$, $p = 0.23$ for L7/GluA3-KO vs. control littermates gain values).

(D) Oculomotor adaptation was assessed through paradigms aiming to either increase (i.e. gain-up paradigm, in which the visual stimulus moves with the same amplitude as the vestibular stimulus, but with opposite direction, resulting in improved VOR compensation) or decrease (i.e. gain-down paradigm, in which both visual and vestibular stimuli move with the same amplitude in the same direction, resulting in cancelation of compensatory eye movements) the amplitude of the VOR. Schematic drawings of the training stimuli are shown in the left column. Our results show that whereas GluA1-KO mice show a comparable increase ($p=0.23$ for final catch trials) and decrease ($p=0.11$) of gains compared to those in WT mice, GluA3-KO mice show significantly impaired gain-up ($p=0.009$) as well as gain-down ($p=0.001$) paradigms. The deficits observed in the global GluA3-KO mice were also present in the PC-cell specific KO (L7/GluA3-KO compared to WT littermates, $p = 0.006$ and $p = 0.04$ for gain-up and gain-down, respectively).

Error bars indicate SEM, * indicates $p<0.05$.

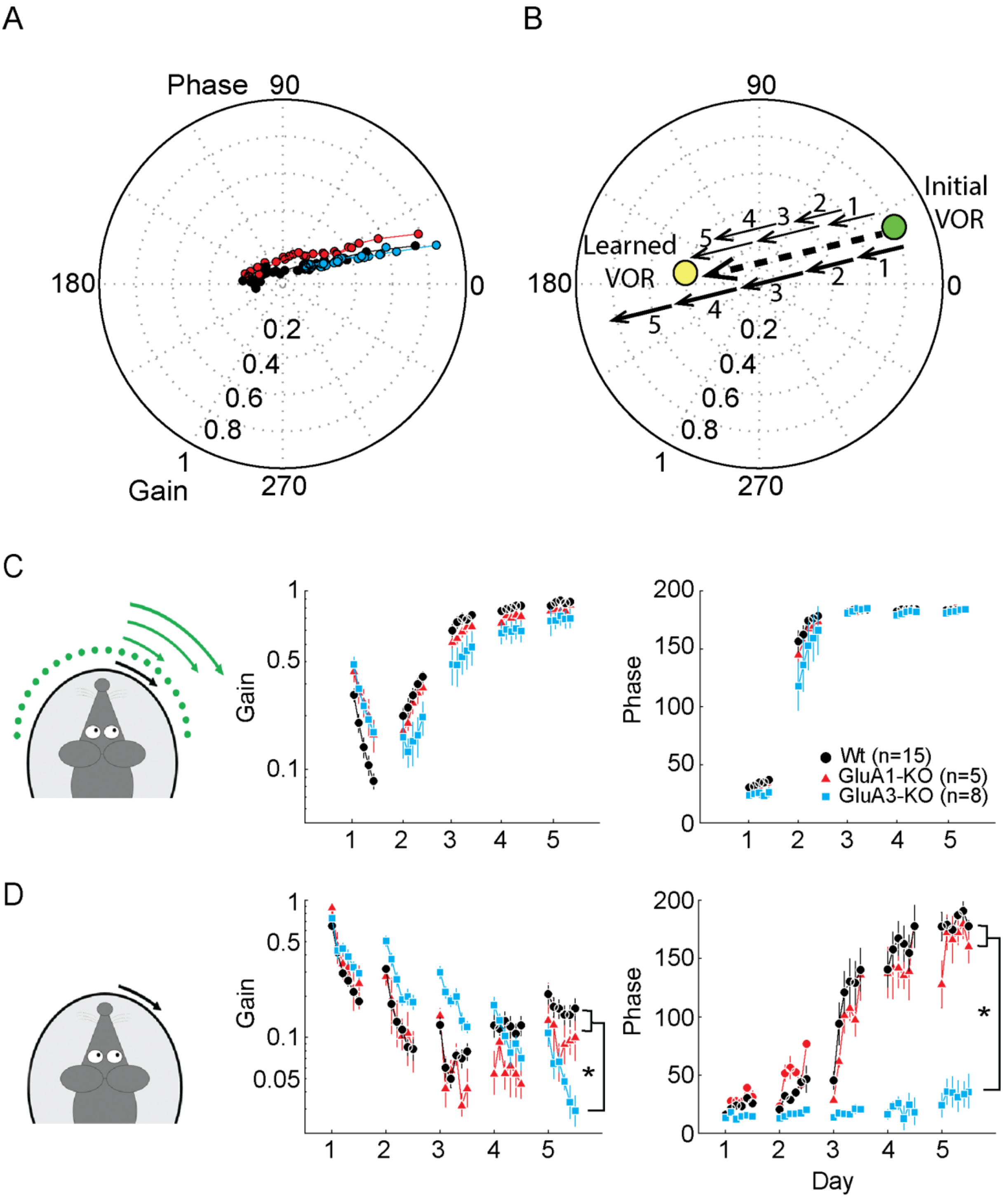

Figure S2. Related to Figure 1, (A) The full range of the 2 variables that explain ocular movements (0 to 1 for the gain and 0 to 360 for the phase) determine a circular bi-dimensional Cartesian plane (shown as a polar plot), in which every eye movement can be defined. Given that phase-reversal learning takes place through a defined common learning trajectory over several days during which phase covariates with gain (left polar plot), we performed statistics on the Cartesian coordinates defining gain and phase using the paired Hotteling's T2-Test.

(B) Polar plots of gain and phase vectorial representation during phase-reversal VOR learning data to illustrate the data analysis procedure. The data are composed of 5 individual learning vectors (one per day) moving across a constant learning trajectory towards the target set by the training paradigm (Phase of 180 degrees; Gain of 1). Based on the raw gain and phase data (A), we first calculate the learning extent for each mouse as the vectorial difference between the final performance and the initial performance (recording 6 of day 5 – recording 1 of day 1) and subsequently average these values per group. Between days of training there is partial retention of motor memories; to calculate the overall consolidation we calculate the ratio between the learning extent and the absolute summed extent of the learning vectors as if there was no memory loss overnight (100% consolidation). This ratio calculated per mouse is then also averaged across the mice, generating consolidation values for each group.

(C) Eye movement behavior of 4-6 week old GluA3-KO mice is virtually identical to that of 3-5 month old mice. Scatter plots of gain and phase values of 4-6 week old mice during the visuo-vestibular training for VOR phase-reversal shows no significant differences in the ability to follow the training signal ($p>0.05$ for last training recording on day 5 for comparison of GluA1-KO vs GluA3-KO and of WT vs GluA3-KO).

(D) Scatter plots of gain and phase values of the VOR catch trials show that WT and GluA1-KO mice, but not GluA3-KO mice, are able to reverse the phase of the VOR after training ($p<0.01$ for last catch recording on day 5 for comparison of GluA1-KO vs GluA3-KO and of WT vs GluA3-KO). For comparison with data in 3-5 month old animals see also Figure 1.

Error bars indicate SEM, * indicates $p<0.05$.

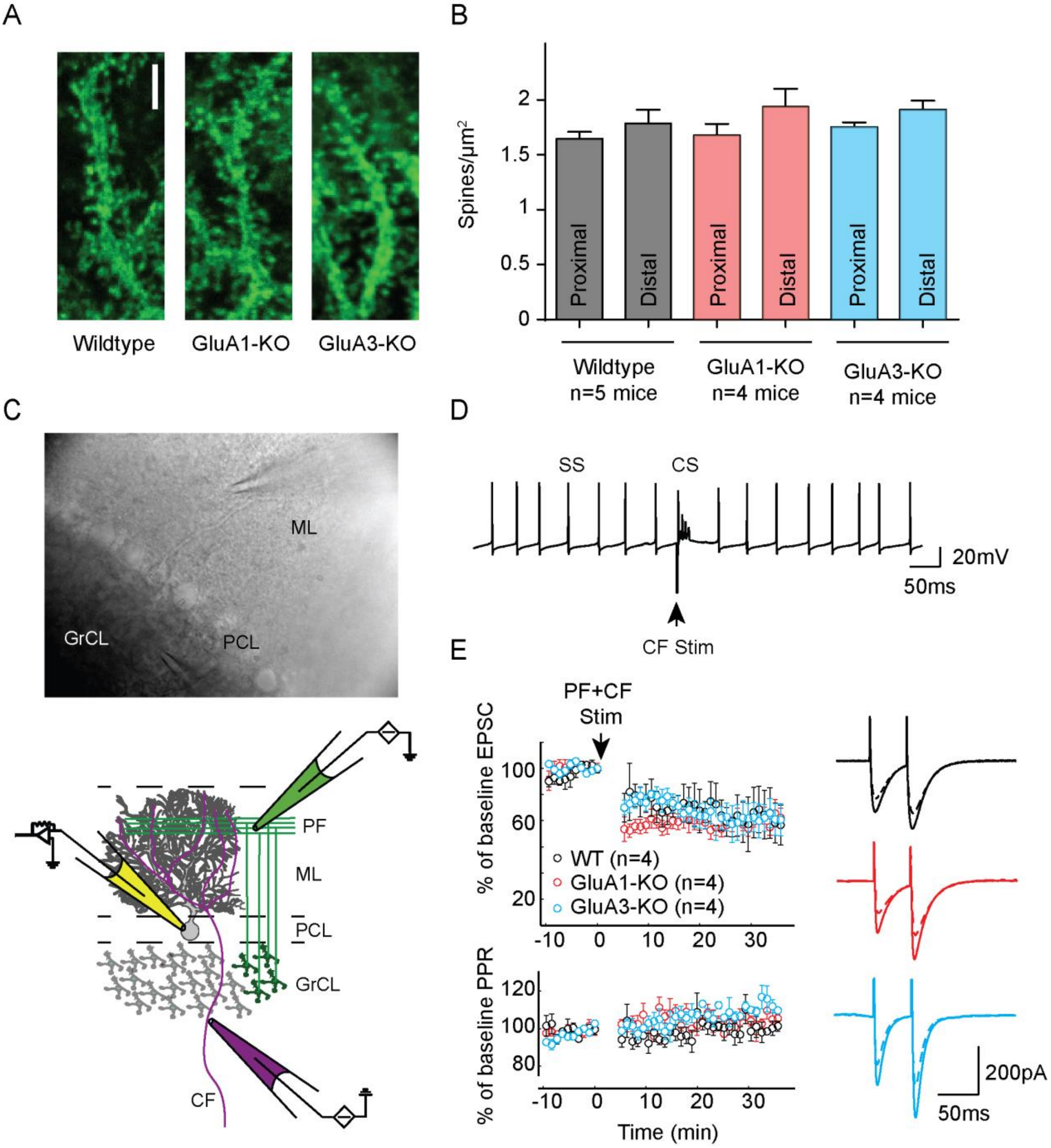

Figure S3. Related to Figure 2, PCs lacking GluA1 or GluA3 have comparable spine density and show comparable levels of LTD induction compared to those in wild type (WT) PCs.

(A) Representative confocal optical planes (0.5 µm thick) of PC distal dendrites used to quantify spine densities of WT, GluA1-KO or GluA3-KO PCs.

(B) Densities plotted for each genotype correspond to the average spine density of at least 20 dendritic branches of PCs in lobules V to X per animal. Scale bar = 5 µm. The results show that neither the lack of GluA1 nor that of GluA3 yielded differences in spine densities of proximal or distal dendrites of PCs.

(C) Scheme of cerebellar cortical circuitry (bottom panel) and representative picture of the in-vitro preparation (top panel) showing positions of recording electrode (yellow) at PC soma and stimulus electrodes (green and purple) at parallel fiber (PF) beam and climbing fiber (CF), respectively. ML, PCL and GrCL indicate molecular layer, Purkinje cell layer, and granule cell layer, respectively.

(D) PCs were recorded in current clamp mode and the location of the stimulus pipettes were determined functionally by evoking responses to electrical stimulation at resting potential. Once the proper locations were identified, cells were kept in hyperpolarized state (-80 mV approx.) and a conjunctive CF and PF stimulation protocol was applied to the cell for 5 minutes (see Suppl. Methods for details).

(E) Both GluA1-KOs (red) and GluA3-KOs (blue) show similar cerebellar synaptic weakening after LTD induction (top panel) compared to WT littermates (black) with unchanged PPR over time (bottom panel). Representative traces of paired EPSCs before (solid lines) and after LTD induction (dashed lines) (right panels; genotypes match the color codes in B).

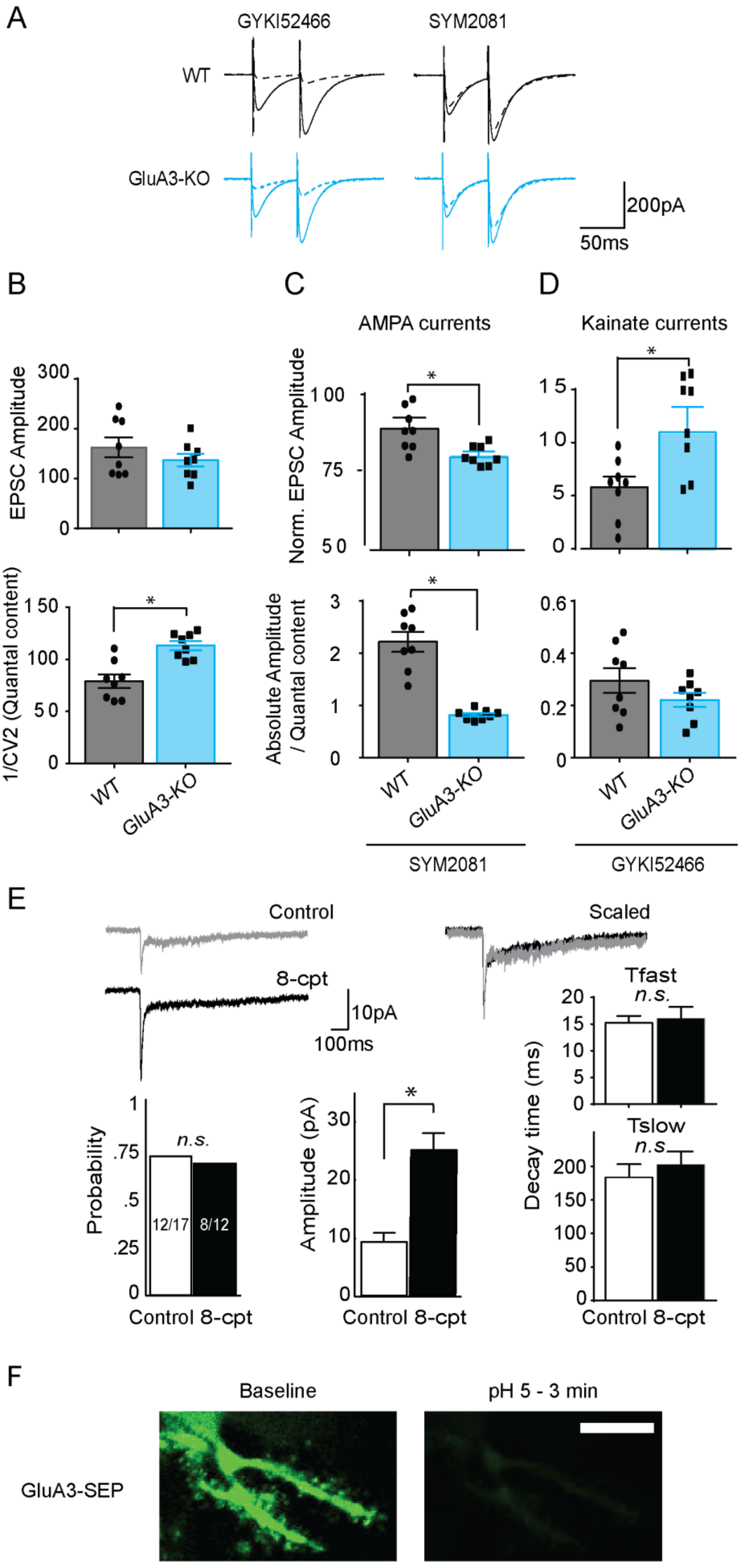

A
GYKI52466
SYM2081
WT
GluA3-KO
200pA
50ms
B
C
D
AMPA currents
Kainate currents
EPSC Amplitude
300
200
100
0
Norm. EPSC Amplitude
100
75
50
*
15
10
5
0
1/CV2 (Quantal content)
150
100
50
0
Absolute Amplitude / Quantal content
3
2
1
0
0.6
0.4
0.2
0
WT
GluA3-KO
SYM2081
GYKI52466
E
Control
8-cpt
Scaled
10pA
100ms
Tfast
n.s.
20
15
10
5
0
Decay time (ms)
Tslow
200
150
100
50
Probability
1
.75
.5
.25
n.s.
12/17
8/12
Amplitude (pA)
30
20
10
Control 8-cpt
F
Baseline
pH 5 - 3 min
GluA3-SEP

Figure S4. Related to Figures 2, 4 and 6. Kainate receptors do not compensate for weakening of glutamatergic transmission at PF to PC synapses in the absence of GluA3.

(A) To assess possible compensatory components in the glutamatergic transmission of PCs in GluA3-KO mice we investigated the impact of blocking either AMPA-receptors with 30 µM of GYKI-52466 (Cossart et al., 2002) or kainate receptors with 5 µM of SYM2081 (Yan et al., 2013) after establishing a stable baseline of eEPSCs in WT and GluA3-KO PCs.

(B) PF stimulation intensity was manually adjusted to obtain comparable EPSC amplitudes between 100-200 pA in WT and GluA3-KO PCs ($p = 0.3$, GluA3-KO vs. WT). The average quantal content released to produce events of comparable amplitude (estimated as the inverse of the square coefficient of variation; Kerchner and Nicoll, 2008), was significantly higher in the GluA3-KO ($p < 0.001$), indicating post-synaptic weakening.

(C) Blocking AMPARs reduced the total glutamatergic transmission in GluA3-KO PCs by 89±2%, which was significantly less than that in wild-type PCs (94±2%; $p = 0.01$ for GluA3-KO vs. WT, top panel). However, this difference was exacerbated after normalizing the amplitude to the quantal content, revealing that in the absence of GluA3, PCs have about half the normal magnitude of AMPA-mediated current ($p < 0.001$, bottom panel).

(D) To investigate to what extent kainate receptors can compensate for an impairment in GluA3-dependent transmission in PCs (Yan et al., 2013), we investigated the impact of a blockage of kainate-receptors in both WT and GluA3-KO PCs. The contribution of kainate-receptor mediated events to EPSC amplitude normalized to baseline magnitude was significantly higher in PCs of GluA3-KO (21±1.5%) than that in WT PCs (16±3%; $p = 0.024$ for GluA3-KO vs. WT, top panel). However, when normalized to the quantal content, the absolute contribution of kainate receptors was comparable among genotypes ($p=0.19$, bottom panel). Together, these data indicate that glutamatergic transmission in GluA3-KO mice can be largely explained by GluA1/GluA2-mediated AMPA-currents and to a lesser extent by kainate-currents, none of which is able to compensate for the synaptic weakening caused by the absence of GluA3.

(E) Excised patches of PC somata that received puffs of 100 µm AMPA generated significantly larger currents when 8-CPT-2Me-cAMP (8-CPT) was present in the internal solution. Note that the control

patches showed the same probability of presenting AMPA events (left). Fast desensitizing and slow decay time kinetics were also unchanged (right panels).

(F) Super-ecliptic pHluorin (SEP) fused to GluA3 AMPARs showed the expected pH sensitivity. Washing in of acidic ACSF (pH 5) produced a dramatic reduction in the fluorescence intensity of externalized GluA3-SEP receptors. This is in line with the fact that GluA3-SEP AMPARs internalized in acidic vesicles contribute marginally to the fluorescent signal imaged. Scale bar, 200 µm.

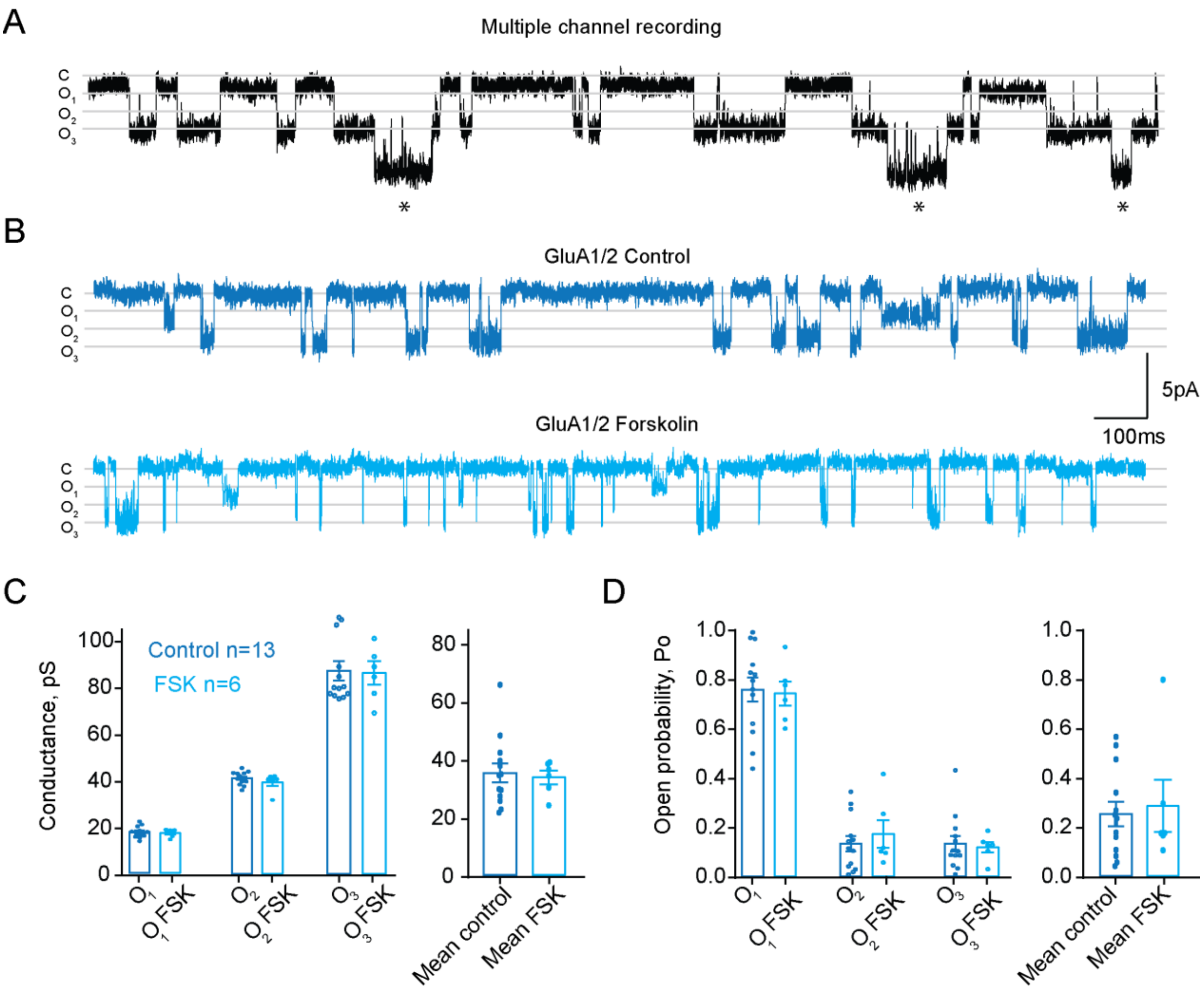

Figure S5. Related to figure 5, GluA1-containing AMPARs single channel properties are unchanged after forskolin application. (A) Example of multichannel activity recording. The presence of “escalated” openings that produced multiple conductance levels (asterisks) before reaching baseline was used as a criterion to discard recordings with multiple channels. (B) Single channels of GluA1-containing

AMPARs showed comparable behavior in the presence and absence of forskolin. Note that under baseline conditions (top panel) the conductance level was higher than that of GluA3 channels as presented in main Figure 4A (top panel). (C) Conductance of the 3 different open levels of these channels was unchanged in the presence of forskolin and also comparable to that of GluA3 channels. (D) The relative fraction of openings and overall open probability of GluA1 channels was also unchanged after forskolin application and it resembled that of cAMP-activated GluA3 channels.

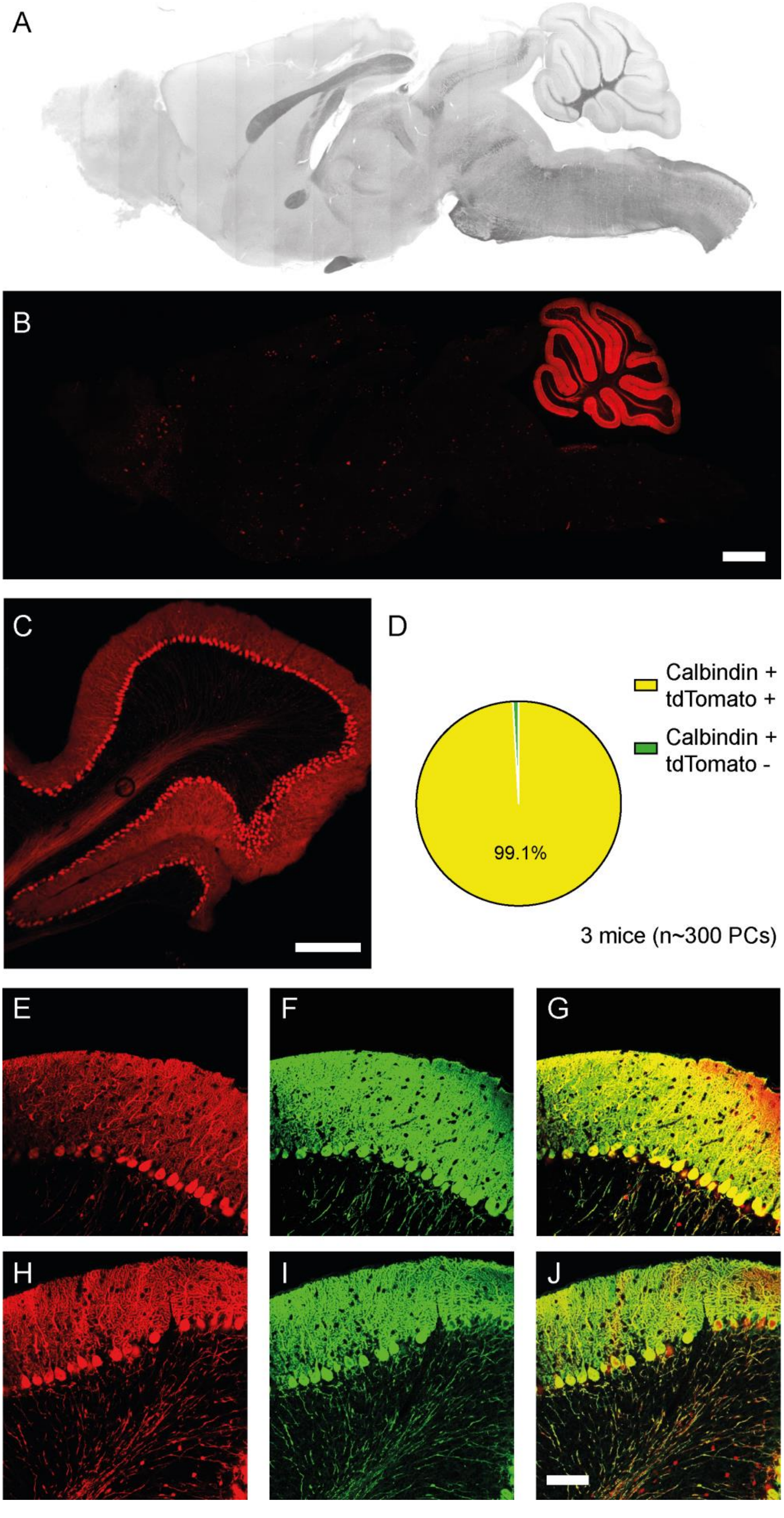
A
B
C
D
Calbindin +
tdTomato +
Calbindin +
tdTomato -
99.1%
3 mice (n~300 PCs)
E
F
G
H
I
J

Figure S6. Related to Figure 8, Cre-dependent tdTomato expression under the L7 promotor confirms its Purkinje cells specificity. (A) Example of a L7Cre/floxedGluA3-KO mouse sagittal brain slice in bright field. (B) Same brain slice imaged with an epifluorescence microscope reveals how tdTomato expression is restricted to cerebellar PCs. (C) PCs in the vestibulocerebellum (flocculus and paraflocculus) also express the reporter under the L7 promotor. (D) Quantification of the population of PCs expressing tdTomato under the L7 promotor. Nearly all PC's with tdTomato (E, H) express calbindin (F, I with single labeling in green; G, J with double labeling in yellow) and vice versa, proving that the L7 promotor can be effectively used to genetically manipulate virtually the entire population of PCs. Scale bars 1 mm (A,B), 250 µm (C) and 100 µm (E-J).

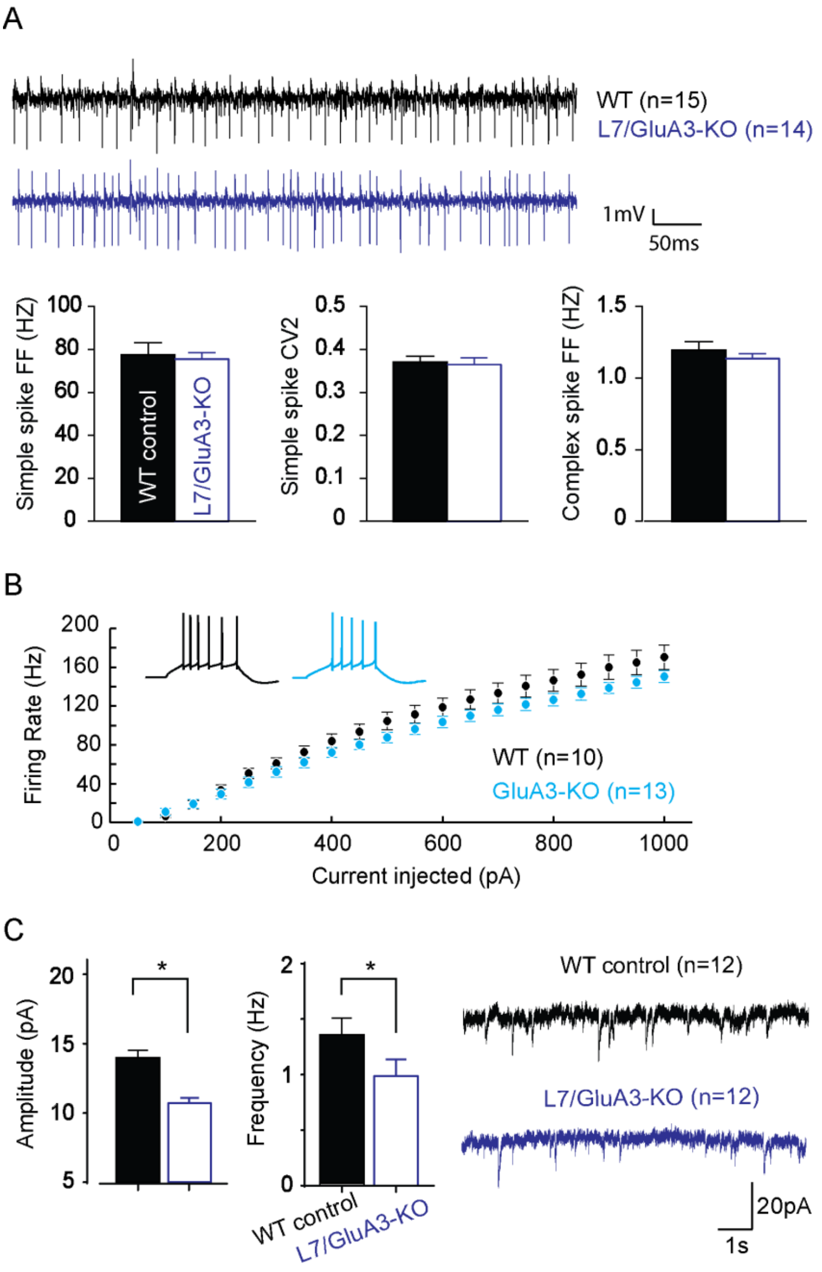

Figure S7. Related to Figure 8, GluA3 lacking PCs show intact excitability in-vitro and in-vivo despite their reduced synaptic transmission. (A) In-vivo spontaneous firing of L7/GluA3-KO PCs show comparable firing frequency and regularity of simple spikes as well as comparable amount of complex spikes, suggesting once more that, despite weaker PF to PC synapses, PC excitability is unaffected. (B) Short square steps of increasing current injected into PCs of both wild-types and GluA3-KOs showed no differences in the I/V relationships between genotypes ($F(1,21)=2.3$, $p = 0.14$, Repeated Measures ANOVA), showing that despite the weaker synaptic transmission in the absence of GluA3, PCs have unchanged excitability in vitro. (C) Synaptic transmission is also reduced in the PC specific KO for GluA3 (L7/GluA3-KO) tested in-vitro. Error bars indicate SEM, * indicates $p<0.05$.

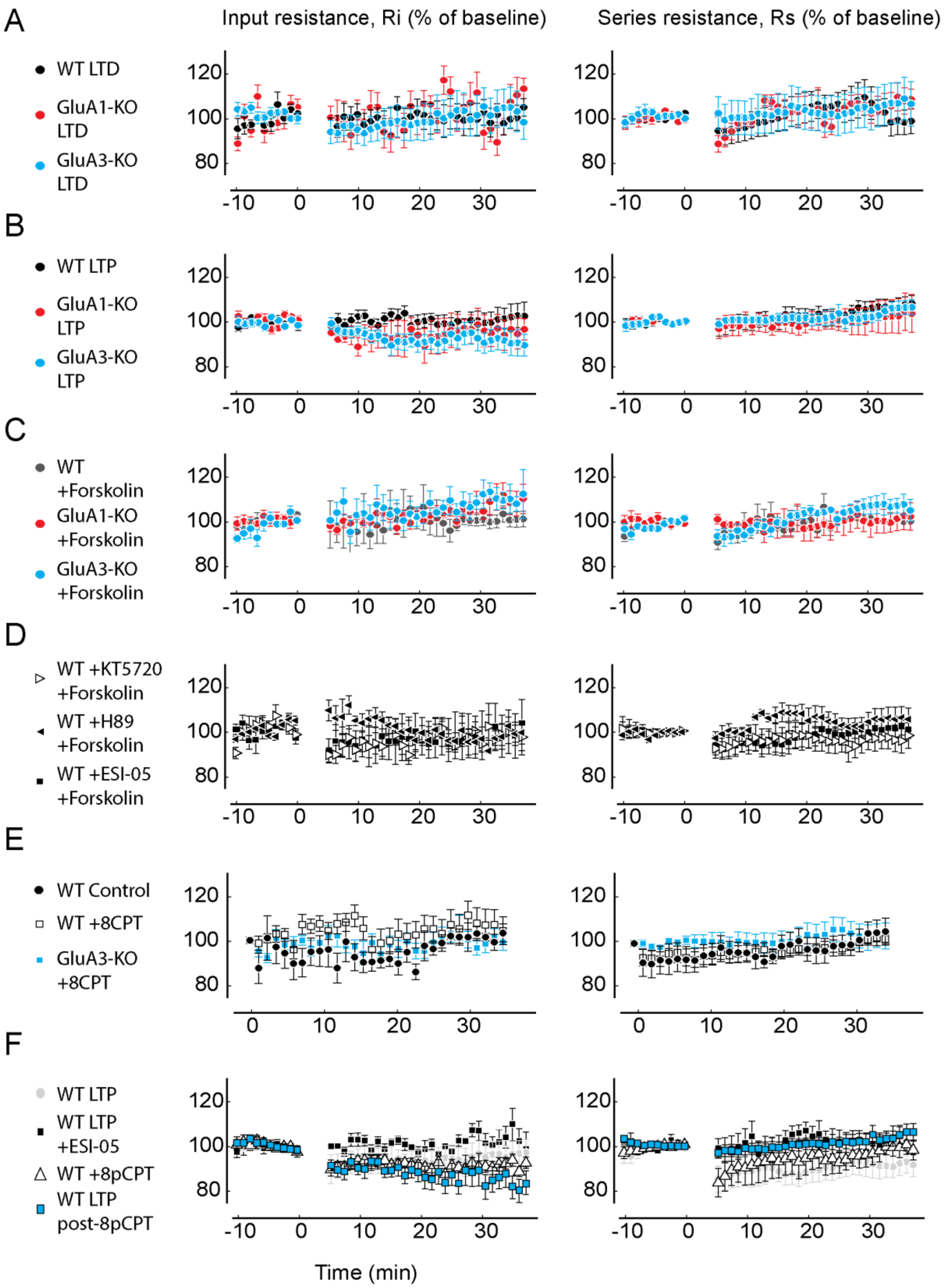

Figure S8. Related to Figures 2, 3, 6 and 7. Overview of membrane resistance (Rm) and series resistance (Rs) of every group of PCs used to generate the experimental figures of the current study. Data are plotted with the same color code as in main figures. All PCs that had a change in resistance

bigger than 20% over a period longer than 2 minutes were discarded for further analysis. Rs and Rm for data shown in figure 2C (A), figure 2D (B), figure 3A (C), figure 6A (D), figure 6B (E) and figure 7A-B (F). Error bars indicate SEM.

EXTENDED EXPERIMENTAL PROCEDURES

Mice

Inbred breeding colonies were used to obtain the experimental knockout mice. GluA1-KO mice, kindly provided by Dr. R. Huganir (Kim et al., 2005), were generated by mating heterozygous c57bl6/129 mice; GluA3-KO and wild-type littermates were bred from c57bl6x129P2-Gria3tm1Dgen/Mmnc mutant ancestors (MMRRC, Davis, CA) at least 6 times backcrossed to c57bl6 mice; and Purkinje cell specific GluA3 knockout mice were generated by crossing floxed GluA3 mice (Sanchis-Segura et al., 2006) with L7-Cre mice (Barski et al., 2000). All experiments were conducted in line with the European guidelines for care and use of laboratory animals (Council Directive 86/6009/EEC). The experimental protocol was approved by the Animal Experiment Committee (DEC) of the Royal Netherlands Academy of Arts and Sciences (KNAW). All data of the experiments described below were acquired and analyzed in a blinded fashion with respect to the genotype.

Eye movement recordings and oculomotor learning tasks

Baseline performance of compensatory eye movements and VOR adaptation were first tested in three groups of male mice at the age of 4-6 weeks and 3-5 months. These included wild-type littermate mice (WT, n = 15 + 14, for both age categories, respectively), GluA1 knockout mice (n = 5 + 6) and GluA3 knockout mice (n = 8 + 6). Mice were surgically prepared for chronic head restrained experiments (de Jeu and De Zeeuw, 2012). During the experiment the mouse was placed head-fixed in a holder tube on a vestibular motion platform (R2000 ‘Rotopod’, Parallel Robotic Systems Corporation, Hampton, USA). Left eye orientation was measured using video pupil tracking with a table-fixed CCD camera (Pulnix TM-6710CL, 120 frames/s) and IR illumination (850 nm LED, 6.5 cm distance from the eye). Pilocarpine (2%) eye drops were applied before the experiment to limit pupil dilatation in darkness. Online image analysis was performed to extract the location of pupil edges and corneal light reflections using custom built software for Labview (National Instruments, Austin, TX, USA). Angular eye velocity was computed offline using custom software written for Matlab (The Mathworks Inc., Natick, MA) using the algorithm outlined elsewhere (Stahl et al., 2000). Saccadic eye movements and quick-phases of the vestibular nystagmus were removed using a 50°/s velocity threshold and 200 ms margins at each threshold crossing. Each mouse was accustomed to the setup for a period of three training days before the experimental data were collected. The horizontal VOR was characterized in both darkness and light using sinusoidal rotation about the vertical axis, using frequencies ranging

between 1/16th to 4 Hz, presented in a sequence of increasing order, holding constant peak velocity of 18.8°/s. The number of cycles ranged between 5 at 1/16Hz to 60 at 4 Hz. Mice were subjected to a VOR cancellation stimulus on the first day (in-phase sinusoidal movement at 0.6 Hz, 5° amplitude of both the table and the visual surround) and a VOR reversal stimulus on subsequent days (2-5), where the amplitude of the visual surround was increased to 7.5° (day 2) and 10° (days 3, 4, and 5). The amplitude of the turntable remained constant at 5° amplitude (18.8°/s peak velocity). Training sessions consisted of 6 VOR measurements (30 cycles, 50 seconds, in darkness) that were alternated with 5 periods of visuo-vestibular mismatch training (300 cycles, 500 seconds). Apart from the training sessions, animals were kept in total darkness during the consecutive training days. The eye movement response was expressed as gain and phase relative to head movement, which was calculated using multiple linear regression of eye velocity to in-phase and quadrature components of the turntable velocity trace. Gain of the eye movement response was defined as the ratio between the eye velocity and the table velocity magnitudes. Phase was expressed in degrees and offset by 180°, so that a phase of 0° indicates an eye movement that is in-phase with contraversive head movement; positive phase values indicate phase leads. Consolidation of the adapted VOR was assessed by computing the ratio between the long-term change in VOR and the cumulative sum of short-term changes in VOR of preceding training sessions. The long-term change was defined as the absolute difference between the ending VOR on day 5 and the naive VOR on day 1. The short-term change was defined as the absolute difference between the VOR at the beginning and end of a training session. For a period of at least 10 days animals were allowed to rest between different VOR adaptation protocols. Bivariate 2-sample Hotelling's T2-test was used to compare gain and phase values between groups, and One Way ANOVA/ Tukey post-hoc test was used for cumulative consolidation values.

Spine density quantification in Purkinje neurons

In order to calculate the spine density in PCs, 5 WT, 4 GluA1-KO and 4 GluA3-ko mice received an overdose of sodium pentobarbital via IP injection and were perfused intracardially with 10 ml of PB 0.1M (pH 7.6) followed by 60 ml of fixative (4% paraformaldehyde in 0.1 M PB, pH7.6) at a rate of 5.5 ml/min. Brains were carefully removed from the skull, post-fixed for a maximum of 2 hours in the same fixative solution at 4°C, immersed in 30% sucrose in PB at 4°C until they sank, and subsequently cut

into 40µm thick frontal sections, which were collected as four matching series. For calbindin detection, the slices were incubated in blocking solution containing 10% horse serum in 0.1 M PB to minimize non-specific binding of the antibodies. After 1 hour, blocking solution was replaced by the primary antibody solution containing 5% horse serum in 0.1M PB, rabbit anti-Calbindin antibody (Chemicon, Millipore) at a concentration of 1:1000 for 12 hours at 4°C. After several rinses with 0.1 M PB slices were incubated for 4 hours in a solution containing 5% horse serum in 0.1 M PB and horse anti-rabbit combined with Alexa 488 secondary antibody at a concentration of 1:200. After several rinses, slices were mounted and covered with Dako mounting medium (Dako), and imaged under a confocal microscope (Leica SP5). All images were acquired with the same settings and the analysis was performed with ImageJ. Stacks of pictures across the Z-axis (10-30 µm) were made to count total spine number in proximal (max 30 µm away from the PC soma) and distal dendrites of PCs. The spine density was calculated for each dendrite dividing the dendrite's spine count by its length; all images were processed using ImageJ. All proximal and distal dendrites counted were averaged for each mouse and mice of the same genotype were averaged to obtain the final spine densities (Figure S3A-B).

In-vitro electrophysiology

Sagittal slices of the cerebellar vermis (250 to 400 µm thick) from 4 to 6 weeks old mice were obtained in ice-cold oxygenated "slicing" solution containing (in mM) 2.5 KCl, 1 CaCl2, 3 MgCl2, 25 NaHCO3, 1.25 NaH2PO4, 240 sucrose, 25 D-glucose and 0.01 kyneurenic acid. Slices were transferred to the same slicing solution at 34°C for 5-10 minutes and then transferred to oxygenated ACSF at 34°C containing (in mM) 124 NaCl, 5 KCl, 1.25 Na2HPO4, 1 MgSO4, 2 CaCl2, 26 NaHCO3, 20 D-glucose. Subsequently, the slices were allowed to recover for at least 30 minutes until they were moved to the recording chamber containing the same oxygenated ACSF with 100 µM picrotoxin to prevent GABAergic transmission at near physiological temperature of 30±2°C. Whole-cell patch-clamp recordings of Purkinje cells located in lobules VI to X were performed using an EPC-10 amplifier (HEKA, Lambrecht). 3-5 MΩ resistance patch pipettes were filled with (in mM) K-Gluconate 122.5 mM, NaATP 4, NaGTP 0.4, HEPES 10, NaCl, KCl 9 and 0.6 mM EGTA (Sigma) at pH 7.25 for all the recordings that required current clamp mode (including LTP) or with (in mM) 115 mM cesium

methanesulfonate, 20 mM CsCl, 10 mM Hepes, 2.5 mM MgCl2, 4 mM Na2ATP, 0.4 mM Na3GTP, 10 mM sodium phosphocreatine (Sigma), and 0.6 mM EGTA (Sigma), at pH 7.25 for the experiments that were exclusively done in voltage clamp mode. For both voltage-clamp and current-clamp recordings, PC membrane potential was held at -70mV to prevent spontaneous firing. Series resistance (5-10 MΩ) was measured before the experiment and compensated with standard procedures. During the experiment series and membrane resistances were monitored by applying a 100 ms hyperpolarizing pulse (-10 mV). Only cells with stable membrane and series resistance (change < 20% of the last 5 minutes of recordings compared to the last 5 minutes of baseline) were included in the analysis (Figure S8). Whole-cell recordings were digitized at 40 kHz and filtered with a Bessel filter at 4 kHz for voltage clamp recordings (8 kHz for current clamp mode). PF to PC LTD was induced by pairing PF stimulation at 1Hz for 1 minute with a 100 ms somatic depolarization from 70 mV to 0 mV, mimicking climbing fiber input (Linden, 2001; Saab et al., 2012), or by pairing PF stimulation at 1Hz for 5 minutes with real climbing fiber stimulation at 1Hz (Schonewille et al., 2011). Instead, PF to PC LTP was induced by PF stimulation alone at 1 Hz for 5 min. To monitor EPSC amplitude over time, two test responses to a PF pulse (with 50 ms interval) were evoked every 20s in voltage-clamp mode. In LTP experiments, cells were switched to current-clamp mode for tetanization. Paired-pulse ratio (PPR) was calculated as the ratio of the amplitude of the second evoked excitatory postsynaptic current (eEPSC) to that of the first. eEPSC amplitudes and PPR were averaged per minute and normalized for final representation. For the experiments on intrinsic excitability recordings were performed in current-clamp mode, again using an EPC-10 amplifier (HEKA Electronics). Intrinsic excitability was monitored through injection of brief steps (550 ms) of increasing depolarizing current (20 steps from 50 to 1000pA). The spike count was taken as a measure of excitability. Input resistance (Ri) was measured by injection of hyperpolarizing test currents (200 pA; 100 ms) and was calculated from the voltage transient toward the end of current injection. Recordings were excluded if the input resistance varied by > 20%.

Single channel activity was measured in cell attached configuration with pipettes between 6-8 MΩ of resistance, containing the same intracellular solution used for whole cell recordings but containing 100 µM of S-AMPA (Tocris). After reaching a patch resistance above 2 GΩ, the patch voltage clamp was decreased from close to resting potential (-60 mV approx.) to twice as negative (-120 mV approx.). In this configuration the ionic driving force across the channel was reversed and

therefore the openings produced depolarizing events in the patch pipette. To determine the actual driving force across the AMPAR we broke into whole cell mode after the single channel recording was acquired and measured the cell resting potential. The driving potential, resulting from subtraction of the resting potential and clamped voltage, was used to calculate the receptor conductance. To further corroborate that the openings observed were caused by AMPARs, a subset of channels was also recorded close to resting potential voltages (-60 mV) and at 0 mV. When clamped close to cell resting potential, the driving force across the channel was minimal and the openings were no longer visible. When clamped at 0 mV the events detected by the pipette were of similar size, but the driving force was reversed, consistent with AMPARs behavior.

For the outside-out patches of AMPA responses, pipettes with 4-6 MΩ resistance were use to establish a Giga-seal (1 GΩ resistance) with PC somata. After compensating the capacitance artifact, we let the seal rest until it reached a resistance above 2 GΩ. After breaking into whole cell mode, the pipette was slowly retracted until both the cell and the outside-out patch were re-sealed again. Every 20 seconds a 100 ms puff of 100 µM AMPA was delivered with a Picospritzer III (Parker, Hollis, USA) to generate an AMPA-dependent response. In each sweep, a 100 ms depolarizing test pulse (-10 mV) was applied in order to test series resistance and membrane capacitance. Only patches with a constant resistance over 1 GΩ were considered for analysis. Membrane capacitance was used to control for outside-out patch size, assuming a specific membrane capacitance of 0.01 pF per 1 µm2 (Schmidt-Hieber and Bischofberger, 2010). Our patches presented comparable estimated areas of 12.1±0.9 and 11.8±0.8 µm2 in control and 8-CPT containing patches, respectively (p=0.42).

Drugs and pharmacology

For mEPSC recordings, tetrodotoxin (TTX, 1 µM, Sigma) was added to the bath solution to block network activity in order to only measure excitatory spontaneous release. In order to isolate the specific contribution of AMPA and kainate receptors to glutamatergic transmission in WT and KO mice, the AMPA specific blocker GYKI52466 (30 µM, Sigma) or the kainate specific blocker SYM2081 (5 µM, Sigma) were added to the extracellular bath solution. For pharmacological investigation of the cAMP-GluA3 dependent pathway the following membrane permeable drugs were added to the bath of ACSF: 50 µM Forskolin (adenylyl cyclase activator, Sigma), 20 µM H89 (PKA antagonist, Tocris), 5

µM KT5720 (PKA antagonist, Sigma), and 10 µM ESI-05 (EPAC antagonist, BioLog). In addition, we applied the membrane non-permeable agonist for EPAC, 8-CPT-2Me-cAMP (20 µM, Tocris Bioscience) to the intracellular whole cell recording solution to investigate the postsynaptic impact of EPAC. In order to obtain a monophasic time decay of the AMPA-evoked responses in outside-out patches we added a final mixture of 80 µM PEPA (AMPAR flop splice variant desensitization blocker, Tocris bioscience) and 100 µM cyclothiazide (CTZ, AMPAR flip splice variant desensitization blocker, Tocris bioscience) to the bath solution.

Analysis of cell physiological data

Spontaneous mEPSC and evoked EPSC recordings were analyzed with MiniAnalysis software (Synaptosoft) and ClampFit (Molecular Devices), respectively. To calculate τ_{fast} (fast desensitizing component) and τ_{slow} (slow non-desensitizing component) of AMPA evoked currents in outside-out patches a double exponential function was fitted using ClamPFit with DC offset set at 0. The decay of the averaged current was fitted to the following equation:

$$I = A_1 e^{-t/\tau 1} + A_2 e^{-t/\tau 2}$$

In this equation τ1 represents τ_{fast}. The percentage of the decay represented by the slow component ($\%_{\ slow}$) was calculated by the function A1/(A1+A2), as described elsewhere (Christian et al., 2013). The weighted decay time constant for AMPA evoked currents in outside-out patches in the presence of desensitization blockers was calculated by dividing the total charge transfer (in fC) by the peak amplitude (in pA). Non-stationary fluctuation analysis of outside-out patches traces was carried out following previously described methods (Alvarez et al., 2002; Benke et al., 2001; Hartveit and Veruki, 2007). In short, peak aligned AMPA evoked currents recorded over 10-15 sweeps per outside-out patch were binned in 10 equally sized bins of 150 ms each and for each bin the mean amplitude and variance were calculated. The data distribution resulting after plotting amplitude versus variance was fitted with the following equation:

$$\sigma^2 = iI - \frac{I^2}{N} + \sigma_b^2$$

Where the variance (σ^2) of the amplitude of the current (I) obtained at each time point is explained as a function of the single unitary current (i) and the number of functional conducting channels (N) with an offset given by the variance of the baseline noise (σ^2_b). The number of funtional channels was extracted from the derivative at I = 0, and the single channel conductance was calculated by dividing the unitary current by the applied voltage with respect to the reversal potential ($V_{holding}$-$E_{reversal}$, -70 mV and 0 mV, respectively). The peak open probability (P_o), which corresponds to the fraction of available functional channels open at the time of the peak current (I_{peak}), was calculated from the following equation:

$$P_0 = I_{peak}/N_{max}$$

In this equation N_{max} represents the theoretical maximum of available channels opened at the point where the theoretical maximum amplitude reaches the minimum variability (σ^2_b) in the given parabola fit.

Single channel activity was analyzed using ClampFit (Molecular Devices). Three detection thresholds were used to detect O1 (1.5 pA), O2 (3 pA) and O3 (4.5 pA) openings in single channel AMPA receptors in steady baseline recordings (no holding current fluctuations). Events with a latency shorter than 0.3 ms were ignored to prevent noise to be recognized as openings.

Statistics

For statistical analysis of behavioral and in-vitro electrophysiological data we used either Matlab statistical toolbox (The MathWorks Inc., Natick, MA, 2000) or GraphPad Prism 6 (La Jolla, California, USA). Although Matlab always reports exact p-values, GraphPad Prism 6 does not report exact values when $p < 0.0001$. Thus we have reported exact p-values when possible, taken into consideration the limitation explained above.

In-vitro two-photon imaging

Organotypic cerebellar slices were made from P7-9 mice using a protocol adapted from previous studies (Hurtado de et al., 2011; Stoppini et al., 1991) and kept in culture 4-7 days prior to the experiments. Slices were then transfected with sindbis virus expressing rat flip GluA3 AMPAR fused to

the pH sensitive version of GFP Super Eccliptic pHluorophor (GluA3-SEP) for a period of 24-48 hours prior to the imaging session. Electrophysiological recordings of PC mEPSCs were performed in this preparation. In our hands, mEPSC amplitudes and frequencies were consistently higher in organotypic cultured PCs than in acute (e.g. Fig 2B and 4B, WT-Acute vs. WT-Organotypic $p = 0.0002$ and $p < 0.0001$ for amplitude and frequency, respectively), using the same concentration of TTX and PTX. For imaging, slices were transferred from the incubation solution to the recording chamber containing ACSF (same composition as mentioned before but with 4 µM calcium and 4 µM magnesium). Three-dimensional images were collected on a custom-built two-photon microscope based on a Fluoview laser-scanning microscope (Olympus). The light source was a mode-locked Ti:sapphire laser (Chameleon, Coherent) tuned at 850 nm using a 60x objective. Optical sections were captured every 0.5 µm from transfected PC dendrites. Fluorescence intensity was quantified from projections of stacked sections using ImageJ software (NIH). For single spine bleaching in the FRAP experiments, a ROI was selected covering the surface of a single spine, which was used to target the laser for 20-30 seconds (with the same intensity as for regular imaging).

In-vivo electrophysiology

Mice (males, 4-6 month old) were prepared for chronic experiments as described previously (Wulff et al., 2009). In short, under general anesthesia a pedestal with a magnet was placed on the frontal and parietal bones of the animal, and a recording chamber was constructed around a small craniotomy in the left occipital bone. After 2 days of recovery, animals were habituated in the setup for 20 min for two days. During the experiments, the animals were alert and immobilized in a custom restrainer. Extracellular activities were recorded with glass micropipettes filled with 2M NaCl solution and advanced into the cerebellar cortex from the surface of Crus I and II. Electrode signals were filtered, amplified and stored for off-line analyses (Spike2, CED, and Cambridge, UK). PCs were identified by the occurrence of both simple spikes and complex spikes, and single-unit activity was confirmed by a brief pause in simple-spike firing following each complex spike (i.e. climbing fiber pause; see De Zeeuw et al., 2011). The whole field visual stimulation was presented by rotating a cylindrical screen (diameter 63 cm) with a random-dotted pattern (each element 2°) at 0.6 Hz with an amplitude of 5°. Offline analysis was conducted in Matlab (Mathworks, Natick, MA, USA). CV2 of simple spikes was calculated as the mean value of $(2 \times (ISIn+1 - ISIn))/ (ISIn+1 + ISIn)$ (Wulff et al., 2009). Modulation of

simple spikes and complex spikes was calculated as the amplitude of the sine wave fitted to the histogram of spike rate. Statistical analysis was done using Student's t-test with SPSS (IBM Corporation, Armonk, NY, USA).

Reference List

Alvarez,O., Gonzalez,C., and Latorre,R. (2002). Counting channels: a tutorial guide on ion channel fluctuation analysis. Adv. Physiol Educ. *26*, 327-341.

Benke,T.A., Luthi,A., Palmer,M.J., Wikstrom,M.A., Anderson,W.W., Isaac,J.T., and Collingridge,G.L. (2001). Mathematical modelling of non-stationary fluctuation analysis for studying channel properties of synaptic AMPA receptors. J. Physiol *537*, 407-420.

Christian,C.A., Herbert,A.G., Holt,R.L., Peng,K., Sherwood,K.D., Pangratz-Fuehrer,S., Rudolph,U., and Huguenard,J.R. (2013). Endogenous positive allosteric modulation of GABA(A) receptors by diazepam binding inhibitor. Neuron *78*, 1063-1074.

Cossart,R., Epsztein,J., Tyzio,R., Becq,H., Hirsch,J., Ben-Ari,Y., and Crepel,V. (2002). Quantal release of glutamate generates pure kainate and mixed AMPA/kainate EPSCs in hippocampal neurons. Neuron *35*, 147-159.

de Jeu,M., and De Zeeuw,C.I. (2012). Video-oculography in mice. J. Vis. Exp. e3971.

Hartveit,E., and Veruki,M.L. (2007). Studying properties of neurotransmitter receptors by non-stationary noise analysis of spontaneous postsynaptic currents and agonist-evoked responses in outside-out patches. Nat. Protoc. *2*, 434-448.

Hurtado de,M.T., Balana,B., Slesinger,P.A., and Verma,I.M. (2011). Organotypic cerebellar cultures: apoptotic challenges and detection. J. Vis. Exp.

Kerchner,G.A., and Nicoll,R.A. (2008). Silent synapses and the emergence of a postsynaptic mechanism for LTP. Nat. Rev. Neurosci. *9*, 813-825.

Linden,D.J. (2001). The expression of cerebellar LTD in culture is not associated with changes in AMPA-receptor kinetics, agonist affinity, or unitary conductance. Proc. Natl. Acad. Sci. U. S. A *98*, 14066-14071.

Saab,A.S., Neumeyer,A., Jahn,H.M., Cupido,A., Simek,A.A., Boele,H.J., Scheller,A., Le,M.K., Gotz,M., Monyer,H., Sprengel,R., Rubio,M.E., Deitmer,J.W., De Zeeuw,C.I., and Kirchhoff,F. (2012). Bergmann glial AMPA receptors are required for fine motor coordination. Science *337*, 749-753.

Schmidt-Hieber,C., and Bischofberger,J. (2010). Fast sodium channel gating supports localized and efficient axonal action potential initiation. J. Neurosci. *30*, 10233-10242.

Stahl,J.S., van Alphen,A.M., and De Zeeuw,C.I. (2000). A comparison of video and magnetic search coil recordings of mouse eye movements. J. Neurosci. Methods *99*, 101-110.

Stoppini,L., Buchs,P.A., and Muller,D. (1991). A simple method for organotypic cultures of nervous tissue. J. Neurosci. Methods *37*, 173-182.

Yan,D., Yamasaki,M., Straub,C., Watanabe,M., and Tomita,S. (2013). Homeostatic control of synaptic transmission by distinct glutamate receptors. Neuron *78*, 687-699.
